# The Complete Mitochondrial Genomes of Six Species of *Tetranychus* Provide Insights into the Phylogeny and Evolution of Spider Mites

**DOI:** 10.1371/journal.pone.0110625

**Published:** 2014-10-16

**Authors:** Da-Song Chen, Peng-Yu Jin, Kai-Jun Zhang, Xiu-Lei Ding, Si-Xia Yang, Jia-Fei Ju, Jing-Yu Zhao, Xiao-Yue Hong

**Affiliations:** 1 Department of Entomology, College of Plant Protection, Nanjing Agricultural University, Nanjing, Jiangsu, China; 2 Department of Entomology, College of Plant Protection, Southwest University, Chongqing, China; Laboratoire Arago, France

## Abstract

Many spider mites belonging to the genus *Tetranychus* are of agronomical importance. With limited morphological characters, *Tetranychus* mites are usually identified by a combination of morphological characteristics and molecular diagnostics. To clarify their molecular evolution and phylogeny, the mitochondrial genomes of the green and red forms of *Tetranychus urticae* as well as *T. kanzawai*, *T. ludeni*, *T. malaysiensis*, *T. phaselus*, *T. pueraricola* were sequenced and compared. The seven mitochondrial genomes are typical circular molecules of about 13,000 bp encoding and they are composed of the complete set of 37 genes that are usually found in metazoans. The order of the mitochondrial (mt) genes is the same as that in the mt genomes of *Panonychus citri* and *P. ulmi*, but very different from that in other Acari. The J-strands of the mitochondrial genomes have high (∼84%) A+T contents, negative GC-skews and positive AT-skews. The nucleotide sequence of the *cox1* gene, which is commonly used as a taxon barcode and molecular marker, is more highly conserved than the nucleotide sequences of other mitochondrial genes in these seven species. Most tRNA genes in the seven genomes lose the D-arm and/or the T-arm. The functions of these tRNAs need to be evaluated. The mitochondrial genome of *T. malaysiensis* differs from the other six genomes in having a slightly smaller genome size, a slight difference in codon usage, and a variable loop in place of the T-arm of some tRNAs by a variable loop. A phylogenic analysis shows that *T. malaysiensis* first split from other *Tetranychus* species and that the clade of the family Tetranychoidea occupies a basal position in the Trombidiformes. The mt genomes of the green and red forms of *T. urticae* have limited divergence and short evolutionary distance.

## Background

The spider mite genus *Tetranychus* includes 149 species [1], some of which are of cosmopolitan agronomical pests, such as *Tetranychus urticae*. With expanded gene families for ABC genes [2], detoxification and digestion [3] in the genome, *T. urticae* represents one of the most polyphagous arthropod herbivores [1]. The genes of detoxification families have also been reported to transcriptionally respond to the defense chemistry of plants after *T. urticae* adapts to a challenging host (tomato) [4]. *T. urticae* and other tetranychid species feed on apples, citrus, cottons, cucumbers, cucurbits, eggplants, grapes, maize, papayas, peppers, soy, strawberries and tomatoes both in the field and in greenhouses. However, it is difficult to identify the tetranychid species by morphological characters because the potential diagnostic morphological characters are limited and often exhibit great phenotypic flexibility. Classification of the green and red forms of *T. urticae* is also a source of debate. These problems demonstrate the need for combining morphological and molecular approaches to identifying the species [5].

The second internal transcribed spacer (ITS2) of ribosomal DNA can be used as a barcode for distinguishing tetranychid species. For example, the ITS2 sequences of *T. kanzawai* and *T. hydrangea* indicate that they are synonymous species, which was confirmed by cross-breeding experiments [6]. One molecular diagnostic tool that has been developed to overcome the difficulty of morphological identification by the ITS2 sequence is restriction fragment length polymorphism (RFLP) [7], [8]. The PCR-RFLP approach has been used to identify tens of *Tetranychus* species and has the ability to distinguish more species. The 5′ end of the mitochondrial COI gene is extensively used as a barcode to identify *Tetranychus* species and to analyze their phylogenetic evolution. Its higher divergence makes COI suitable for investigating intraspecific variation, but its usefulness for resolving phylogenetic species relationships remains limited [9], [10]. The lack and occasional unreliability of sequences in public databases restricts their use as molecular diagnostic tools [5].

Thus, to clarify the molecular phylogeny of *Tetranychus* species, as well as to provide new DNA barcodes, we decided to compare the whole mitochondrial genomes of the green and red forms of *T. urticae* as well as five other major spider mite pests in China. Most metazoan mitochondrial genomes are circular, have a length of approximately 16 kb and encode 37 genes including 13 protein-coding genes (PCGs), two rRNA genes (rRNAs), and 22 tRNA genes (tRNAs) [11]. The databases presently include the mitochondrial genomes of 37 acarids, including 12 of the superorder Acariformes and 25 of the superorder Parasitiformes (Table S1). Several aspects of the mt-genomes of Acari have been examined, including gene rearrangement [12]–[16], tRNA gene loss [17] and atypical short tRNA [13], [14].

Sequencing these genomes will have other benefits. For example it should provide insights into the molecular evolution of acaricide-resistance genes. The rapid development of acaricide resistance of spider mites is a long-standing problem [18], [19]. Several acaricides have been identified as mitochondrial respiration inhibitors [19]–[21]. Resistance to the acaricide bifenazate has been correlated with mutations in the mitochondrial cytochrome b (*cob*) gene [22], [23]. The genomes will also provide information on gene rearrangements [24]–[30], evolutionary pattern and structure of the control region [31], [32], strand asymmetry in nucleotide composition [33] and RNA secondary structure [34].

## Materials and Methods

### Sample origin and identification

Ethics Statement: No specific permits were required for the collection of spider mites because the spider mite is a pest in agriculture and the location is not privately-owned in any way. The field study did not involve endangered or protected species.

Strains of *T. kanzawai*, *T. ludeni*, *T. malaysiensis*, *T. phaselus*, *T. pueraricola* and the green and red forms of *T. urticae* were collected in the field, separately (Table S2). Mites were reared on a leaf of the common bean (*Phaseolus vulgaris* L.) at 25±1°C, 60% r.h. and under a 16/8 h (light/dark) photoperiod. All the species were classified by morphological characteristics [35] and RFLP analyses [7], [8]. For the RFLP analyses, the ITS2 fragment was amplified by PCR using the *Tetranychus* universal primers, rD02 and HC2 (Table S3) and genomic DNA as template, and digested by five restriction endonucleases (DraI, RsaI, MboII, DdeI and HinfI) (Fig. S1).

### DNA processing

DNA was extracted from individual mites with a Wizard Genomic DNA Purification Kit (Promega) according to the manufacturer's protocol. A fragment of the COI gene was amplified by standard PCR using the *Tetranychus* universal primers, T-CO1-F and T-CO2-R (Table S3). PCR fragments were ligated into pEASY-T3 cloning vector (Beijing TransGen Biotech) and the resulting plasmid DNAs were transformed into competent *Escherichia coli* Trans1-T1 cells provided in the cloning kit. The inserted fragments were sequenced with M13f and M13r primers. Long PCR primers for each species were designed according to the COI fragment sequences (Table S3). The mitochondrial genome was amplified by long PCR in one single fragment according to the manufacturer's rapid PCR protocol. The reaction mixture contained 2 µl PrimeSTAR GXL DNA Polymerase (Takara), 10 µl buffer, 4 µl dNTP mixture (2.5 mM each), 1 µl of each primer (10 mM), 1 µl of DNA and water added to total volume of 50 µl. The cycling conditions were 30 cycles of 98°C for 10 s and 68°C for 5 min. Sequencing libraries for the long PCR fragments were prepared by using a TruSeq DNA Sample Prep Kit (Illumina) following the manufacturer's instructions. Each individual library was tagged for a different multiplexing identifier (MID). The libraries were purified with Certified Low Range Ultra Agarose (Bio-Rad), quantified with a TBS380 fluorometer (Invitrogen), pooled and sequenced using a Miseq V2 Reagent kit to generate pair-end reads (read length: 250 bp). The reads for each sample were sorted by tag sequences and assembled into one contig after trimming the tag adapter sequences. The contigs were assembled with COI sequences respectively to obtain integral genome sequences (NCBI GenBank accession numbers: KJ729017 – KJ729023).

### Annotation and analysis

Protein-coding genes (PCGs) were identified by ORF Finder implemented at the NCBI website using the invertebrate mitochondrial genetic code. The sequences of PCGs that matched *T. urticae* mitochondrial genes submitted previously to NCBI [22] were accepted. The tRNA genes were identified using ARWEN with default parameters [36] and the tRNAscan-SE [37] with a cove cutoff score of 0.1, the tRNA-model set to “EufindtRNA-Cove” and source set to “Mixed”. Other tRNA genes and two rRNA genes (*rrnL* and *rrnS*) were determined by sequence similarity to genes in *Panonychus citri* [13], [23], *P. ulmi* and *T. urticae*. The secondary structure models for tRNA and rRNA genes were constructed by comparison with the published secondary structures for *Dermatophagoides pteronyssinus* [16], *Leptotrombidium pallidum* [38], *Panonychus citri* [13], *Steganacarus magnus* [17] and *Palmaria palmata* [39]. The Map, GC content and GC skew of the mitochondrial genome were drawn with the CGView [40]. The base composition, codon usage, Relative Synonymous Codon Usage (RSCU) values and nucleotide substitution were analyzed with Mega ver. 6 [41]. The secondary structure of the A+T-rich regions (putative control region) were constructed with Mfold Server [42] and RnaViz ver. 2 software [43]. The evolutionary pairwise divergence was estimated with Mega ver. 6 with Kimura 2-parameter model [44]. Multiple alignments of 13 PCGs' amino acid sequences were performed with ClustalW [45] as implemented in Mega ver. 6 and then corrected by eye to ensure that alignment was in agreement with protein coding genes and to minimize the number of uninformative gaps (Appendix S1). The order of the PCGs is *atp6, atp8, cox1-3, cob, nad1-4, nad4l and nad5–6*. The length of the alignment was 2974 amino acids in the final dataset. ProtTest (http://darwin.uvigo.es/software/prottest2_server.html) [46] was used to select the best model of protein evolution. It selected mtREV+G+I+F as the most appropriate model for the combined amino acid dataset under the Bayesian Information Criterion (BIC) [47]. Maximum-likelihood (ML) analysis of the amino-acid dataset was performed with PhyML ver. 3.1 [48] under the mtREV+G+I+F model, and Maximum-parsimony (MP) analysis was performed with Mega ver. 6. Bootstrap percentages (BPs) were calculated with 1000 replications. MrBayes ver. 3.2.2 was used for Bayesian analysis under the mtREV model of amino acid substitution with the nst  = 6 rates  =  invgamma command. Two runs were performed simultaneously, each with four Markov chains (one cold, three heated). The analyses were run for 1,000,000 generations and the trees were sampled every 100 generations. The Markov chain stationarity and run parameter convergence were evaluated with TRACER ver. 1.6. The first 25 percent of the trees were discarded as burn-in with the relburnin = yes burninfrac = 0.25 command. Multiple alignments of whole genomic DNA sequences of the Tetranychoidea were performed with ClustalW as implemented in Mega ver. 6 (Appendix S1). jModelTest Ver. 2.1.4 [49] selected the GTR+I+G model as the best model for the nucleotide sequences of the mt genome. The ML analysis was performed and bootstrapped with 1000 replications with PhyML v. 3.1 with the GTR+I+G model, and MP analysis was performed by using Mega ver. 6. The Markov chains were run for 10,000,000 generations with sampling every 1000 generations for the Bayesian analysis. The first 25 percent of the trees were discarded and the remaining trees were used to calculate Bayesian posterior probabilities.

## Results and Discussion

### General features of mitochondrial genome organization

**Figure 1:**
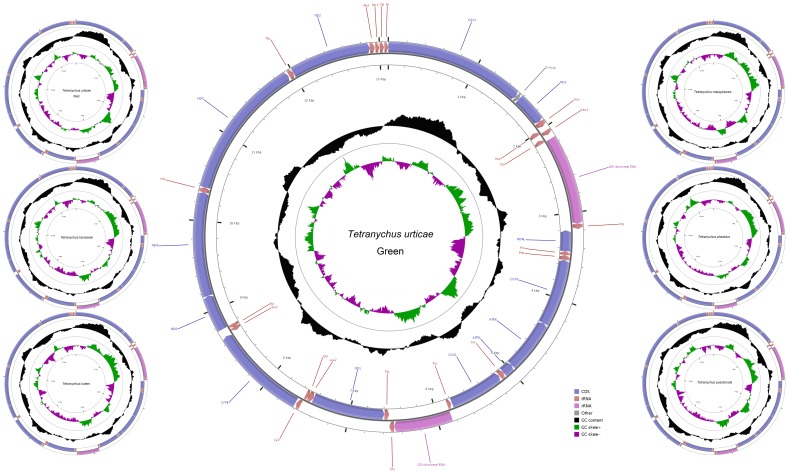
Mitochondrial genome maps of *T. urticae* (green and red forms), *T. kanzawai*, *T. ludeni*, *T. malaysiensis*, *T. phaselus* and *T. pueraricola*. From outer to inner, the 1^st^ circle shows the gene map and tRNA genes are abbreviated by triple letter, with Leu1 = CUN, Leu2 = UUR, Ser1 = AGN and Ser2 = UCN. The 2^nd^ circle shows the GC content and the 3^rd^ shows GC skew calculated as (G−C)/(G+C). GC content and GC skew are plotted as the deviation from the average value of the entire sequence.

The mitochondrial genomes of the seven common tetranychid mites in China are typical circular DNAs (Fig. 1) with lengths of about 13,000 bp (Table S2). To our knowledge, the mitochondrial genome of *T. malaysiensis* is the smallest within all Acari genomes accessible in the GenBank (status March 17, 2014). The mitochondrial genome sizes of the red and green forms of *T. urticae* differ by 4 bp. Thirty-seven genes (13 PCGs, two rRNA genes, and 22 tRNA genes) were identified in each genome (Table S4), which is typical of presently available in metazoan mitochondrial genomes [11]. Twenty genes are encoded on the majority strand (J-strand), whereas the others are encoded on the minority strand (N-strand). The gene order is the same in all seven genomes and the same as that in *Panonychus citri* and *P. ulmi* which are in the same family as *Tetranychus*. However, this gene order is very different from that in other Acari and chelicerates as Yuan's [13] and Van Leeuwen's [23] reports. This suggests that the mitochondrial genome rearrangement event occurred before the divergence of *Panonychus* and *Tetranychus*.

### Base content

**Figure 2:**
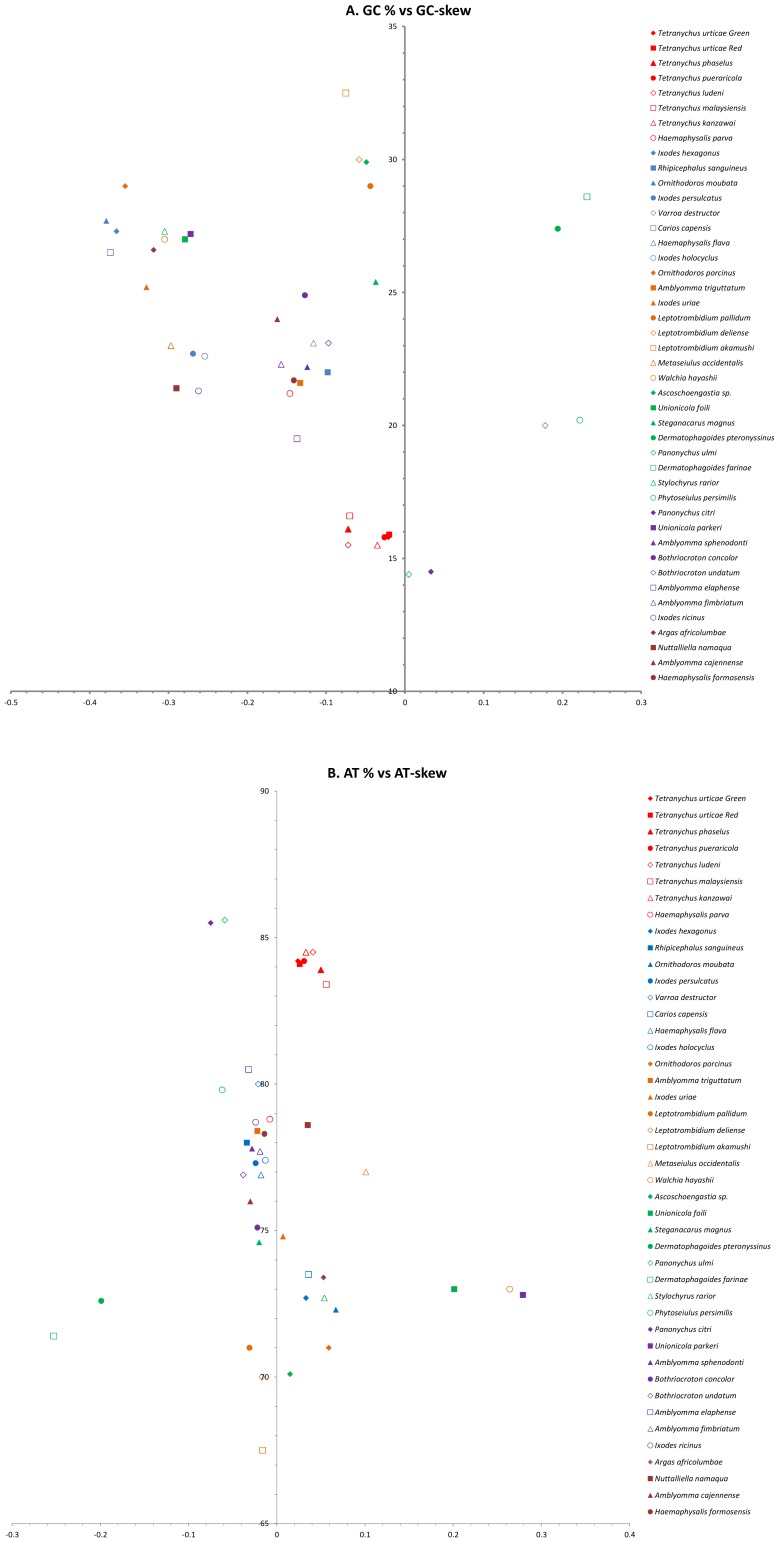
GC% *vs* GC-skew and AT% *vs* AT-skew in the 44 Acari mitochondrial genomes. Values are calculated for mitochondrial genomes of J-strands. The X-axis provides the nucleotide skew values and the Y-axis shows the nucleotide percentages.

The J-strands of the seven mitochondrial DNAs have high A+T contents (83.4–84.5%) which are higher than those for Acariformes (about 74% without the Tetranychidae family) and Parasitiformes (about 77%) (Fig. 2). In Acari, the AT-skew of the mitochondrial genome (average 0.009±0.013) ranges from 0.279 in *Unionicola parkeri* to −0.253 in *Dermatophagoides farina*. Within the genus *Tetranychus*, the species with the highest AT skews in the mitochondrial DNAs are *T. malaysiensis* (0.056), *T. phaselus* (0.050) and *T. ludeni* (0.041). The average GC-skew of Acari mitochondrial genomes is −0.130±0.024, ranging from −0.379 in *Ornithodoros moubata* to 0.231 in *Dermatophagoides farina*. Most Acari have negative mitochondrial genome GC-skews. The exceptions are *Varroa destructor* (0.178), *Dermatophagoides pteronyssinus* (0.194), *D. farinae* (0.231), *Phytoseiulus persimilis* (0.222), *P. citri* (0.033) and *P. ulmi* (0.005). The *Tetranychus* species with the lowest mitochondrial DNA skews are *T. ludeni* (−0.072), *T. phaselus* (−0.072) and *T. malaysiensis* (−0.070). The AT- and GC-skews are quite similar in the mitochondrial genomes of the two *T. urticae* forms. Most metazoan species present a clear strand asymmetry, in which the J-strand is biased in favor of A and C and the N-strand is biased in favor of T and G [50]. The J-strands of the seven mitochondrial DNAs exhibit typical GC-skews, but two completed mitochondrial genomes from the genus *Panonychus* have positive GC-skews. It has been suggested that such reversals are caused by inversions of the A+T-rich regions and replication origin [33].

### Putative control region

**Figure 3:**
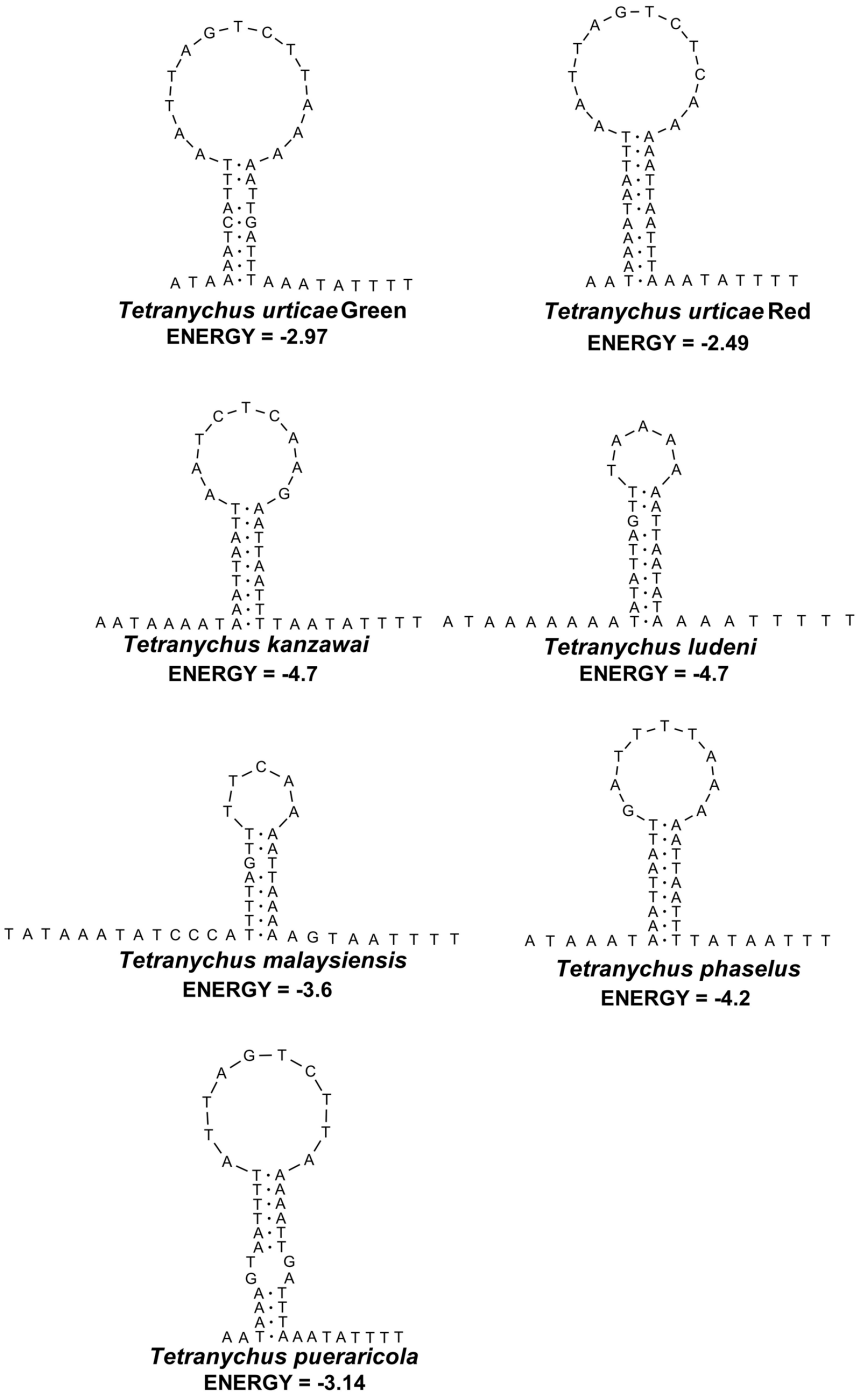
Putative stem-loop secondary structure of A+T-rich regions. The structures were constructed by using Mfold and Watson-Crick bonds are illustrated by black dots. The free energy values (kcal/mol) and the species names are shown below each structure.

The longest non-coding region, which presumably functions as the mitochondrial control region, is 41–44 bp in length and is flanked by the *cox1* and *nad3* genes. Among Acari, the genus *Tetranychus* has the smallest mitochondrial control region. The A+T-rich region is believed to be characterized by a poly-T stretch at the 5′ end, a poly[TA(A)]_n_ stretch close to the poly-T stretch, a stem and loop structure flanked by a TATA motif and a G (A)_n_ T motif [31], [32]. The A+T-rich region in the seven *Tetranychus* genomes can be folded into one stem-loop secondary structure and an apparent poly [TA(A)]_n_ stretch located near the 5′ end of A+T-rich region (Fig. 3). However,none of the hairpin structures is associated with a poly-T stretch or flanked by a TATA or G (A)_n_ T motif. The loops in the secondary structures of *T. ludeni* and *T. malaysiensis* are smaller than they are in other *Tetranychus* species, and the stem-loop secondary structure in *T. urticae* and *T. pueraricola* are located closer to the 5′ end of the A+T-rich region. Although similar poly [TA(A)]_n_ stretches are present near the 5′ end of A+T-rich regions in *T. urticae* (green form: TAAAA; red form: TAAAA) and *T. pueraricola* (TAAA), they were folded as stems in the hairpin structures. The A+T-rich regions of *P. citri* and *P. ulmi* are not only longer (57 and 55 bp, respectively), but they also can be folded into two hairpin structures. Short poly-T stretches were identified in the A+T-rich regions of *P. citri* and *P. ulmi*, but the poly [TA(A)]_n_ stretch, TATA or G (A)_n_ T motif are not present.

### Protein-coding genes and codon usage patterns

The total length of the 13 PCGs in *T. malaysiensis* (10210 bp) is shorter than it is in the other *Tetranychus* mitochondrial genomes (10224–10227 bp). All the PCGs start with typical ATN codons (where N is any nucleotide), as is the case in other metazoan mitochondria (Table S4). Only three of the mitochondrial genes in *T. malaysiensis* (*cox1*, *nad6* and *nad4*) and only one gene in *T. phaselus* (*nad4l*) have ATC start codons. In the other genes, ATA, ATT and ATG start codons are more common. In most cases, a given gene has different start codons in the different mitochondrial genomes. The exceptions are *cox3*, *atp6* and *cox2*, which start with ATG in each genome and *nad2*, which starts with ATA in each genome. Although most PCGs have TAA or TAG stop codons, many of them have incomplete stop codons, such as T or TA. The two forms of *T. urticae* have different start codons for *nad3* and different stop codons for *cox1*. In the codons of the 13 PCGs of the seven genomes, the three codon positions have different nucleotide biases (Fig. S2). The A+T content of the third codon position (about 91%) is higher than the A+T contents of the first and second codon positions (about 82% and 78%, respectively). But the third codon position in the *T. malaysiensis* genome has a lower thymine content (47%) and a higher cytosine content (7%) than do the third codon positions in the others six genomes (in which the average T and C contents are 49% and 5%, respectively).

**Figure 4:**
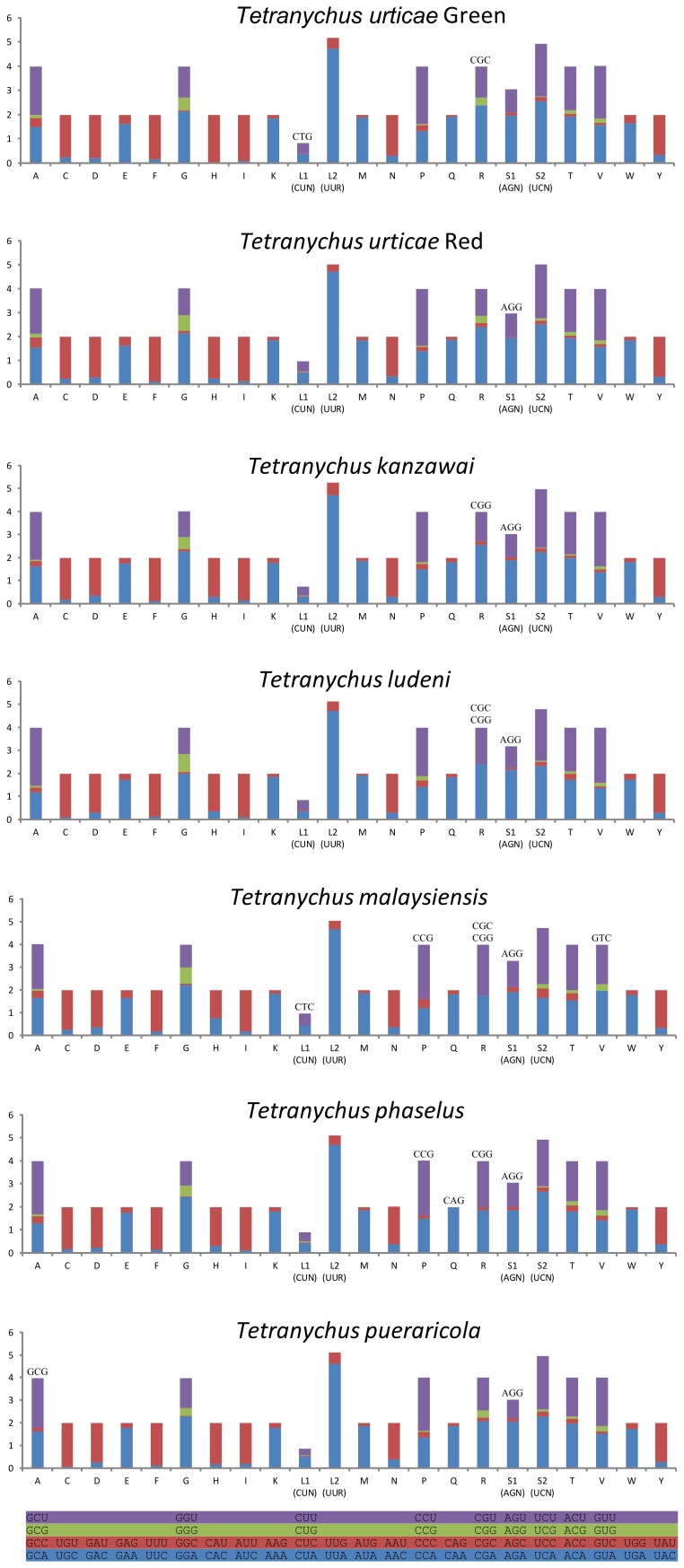
Relative synonymous codon usage (RSCU) for the mitochondrial proteins. The X-axis shows the codon families, while the Y-axis shows RSCU values. Absent codons are shown at the top of the columns.

The amino acid frequencies without start and stop codon are similar between the different *Tetranychus* mitochondrial genomes (Fig. S3). The utmost frequently used amino acids are Phe (14.61–15.26%), Leu (13.14–13.85%), Met (11.25–12.83%), Ile (10.38–11.57%), Ser (9.06–9.65%) and Asn (8.25–8.59%). The mitochondrial genome of *T. malaysiensis* has a slight variety in the amino acid proportion of Met (12.83%, average: 11.77%) and Ile (10.38%, average: 11.29%). The seven AT-rich codons TTT-Phe (13.31–14.35%), ATT-Ile (9.52–11.02%), TTA-Leu (10.30–10.87%), ATA-Met (10.63–12.00%), AAT-Asn (6.71–7.23%), AAA-Lys (4.52–4.84%), and TAT-Tyr (3.48–3.78%) are the most frequently used codons in the *Tetranychus* PCGs. The slightly low ATT codon (9.52%, average: 10.54%) and high ATA codon (12.00%, average: 11.03%) usage in the *T. malaysiensis* mitochondria leads to the variety in the amino acid proportion. A considerable transversion likely occurred between the codon ATT and ATA in the evolution of *Tetranychus* mitochondria. The Relative Synonymous Codon Usage (RSCU) in *Tetranychus* PCGs exhibits a similar pattern and an over-usage of A and T at the third codon positions (Fig. 4). Six codons could not be identified in the *T. malaysiensis* mitochondrial PCGs whereas other *Tetranychus* PCGs abandon only 1–4 codons. Two codons (CTG and CGC) could not be identified in the PCGs of green form of *T. urticae*, while only one codon (AGG) is not present in the PCGs of red form of *T. urticae*. Several arthropods have been reported to translate the codon AGG as lysine instead of serine in mitochondrial genetic codon [51]. However, only one AGG codon was identified in the PSGs of *T. urticae* green form. The other mitochondrial genomes of spider mites including *P. citri* and *P. ulmi* [13] do not use AGG codon. And it is still uncertain which amino acid does the AGG codon in spider mite's mitochondrion translate.

**Figure 5:**
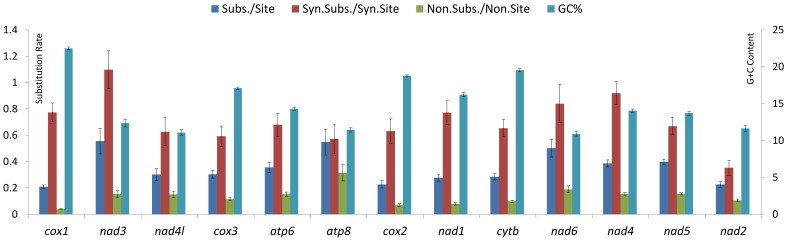
Different evolutionary patterns among protein coding genes of *T. urticae* green form, *T. kanzawai*, *T. ludeni*, *T. malaysiensis*, *T. phaselus* and *T. pueraricola*. Sub./Site, the number of nucleotide substitutions per site from averaging over all sequence pairs, was calculated with JC model. Syn.Subs./Syn.Site, the number of synonymous substitutions per synonymous site (Ks); Non.Subs./Non.Site, the number of nonsynonymous substitutions per nonsynonymous site (Ka); Ks-Ka, the value of Ks minus Ka; the analysis were estimated with Kumar model [68]. The rate variation among sites was modelled with a gamma distribution calculated by Mega ver. 6 and the standard error estimations were obtained by bootstrapping 1000 replicates. Substitution rate is shown on the left Y-axis and G+C content is shown on the right Y-axis.

The evolutionary patterns of the PCGs are different (Fig. 5). The *cox1* gene is commonly used as a taxon barcode because of its high rates of interspecific sequence change and constraints on intraspecific divergence [52], [53]. But this gene exhibits the lowest nucleotide substitution rate per site (0.208±0.014) and lowest value of nonsynonymous substitutions per nonsynonymous site (Ka) (0.04±0.004) compared to the other genes. The nucleotide substitution rate per site (0.555±0.097) and the number of synonymous substitutions per synonymous site (Ks) (1.098±0.143) of *nad3* gene are highest, while the Ka number of *atp8* is highest (0.315±0.062). In addition, the GC content was found to be negatively correlated with both the nucleotide substitution rate per site (R = −0.637, P = 0.019) and Ka (R = −0.715, P = 0.006), while the nucleotide substitution rate per site was found to be positively correlated with Ka (R = 0.825, P = 0.001). Ks was not found to be associated with Ka, GC content or nucleotide substitution rate per site.

### Ribosomal and transfer RNAs

Among the seven *Tetranychus* mitochondrial genomes, the large subunits of rRNA (*rrnL*) are from 982 bp (*T. ludeni*) to 998 bp (*T. urticae* and *T. kanzawai*) in length. The first nucleotide downstream of *trnE* was annotated as the 5′ end of *rrnL* and the first nucleotide upstream of *trnR* was annotated as the 3′ end of *rrnL*. The genes for the small subunit of the rRNA (*rrnS*) are 629 bp (*T. ludeni* and *T. phaselus*) to 645 bp (*T. pueraricola*) in length and are located between *trnY* and *trnG*. Both ribosomal subunits are encoded on the J-strand as is the case in *Metaseiulus occidentalis* [54], *Dermatophagoides farinae* [14], *D. pteronyssinus* [16], *Leptotrombidium pallidum* [55], *P. citri* [13] and *P. ulmi*, whereas the rRNA genes in most species of Acari, arthropods and chelicerates are located on the N-strand [12], [14], [15], [17], [56], [57].

We predicted the *rrnL* and *rrnS* secondary structures of *T. urticae* (Fig S4 and S5). Nucleotides that are identical among the seven *Tetranychus* mitochondrial DNAs are shown in bold in Figures S4 and S5. In contrast, most of the helices found in Acari *rrnL* and *rrnS* genes are present in *T. urticae*. However, four helices (D10, D14, G9 and H3) of *rrnL* and one helix (33) of *rrnS* found in *P. citri* and *L. pallidum* are not present, while *T. urticae* has one more helix (D8) in *rrnL* and three more helices (4, 18 and 22) in *rrnS*. Most ribosomal subunit sequences, especially the 3′-end of both *rrnL* and *rrnS*, are conserved among the seven mitochondrial genomes. The helices D2, D4, D7, D9, and D17 could be identified in all the mitochondrial *rrnL* genes with slight variations, whereas their sequences are not conserved. The sequences of helices 24/25/26 as depicted in *rrnS* are weakly conserved among the genus *Tetranychus*, and the stem-loops in the deduced secondary structures vary in size.

Eleven of the 22 tRNA genes are encoded on the J-strand and the other 11 are encoded on the N-strand. The secondary structures for all tRNAs of the seven mitochondrial genomes were predicted (Table S5). The aminoacyl acceptor stem (AA-arm) and anticodon arm (AC-arm) in the tRNAs are highly conserved among the seven genomes. With a few exceptions among the seven genomes, only seven of the tRNAs (*trnH*, *trnK*, *trnL2*, *trnM*, *trnN*, *trnR* and *trnW*) can be potentially folded into a classical cloverleaf structure, whereas four tRNAs (*trnD*, *trnE*, *trnS1* and *trnS2*) lose the DHU stem (D-arm), eight (*trnA*, *trnC*, *trnF*, *trnG*, *trnL1*, *trnT*, *trnV* and *trnY*) lack the TψC stem (T-arm) and 3 (*trnI*, *trnP* and *trnQ*) appear to lack both the D- and the T-arm. *T. ludeni* differs from the other species in that the D-arm of *trnA* was substituted by a D-replacement loop, and *T. malaysiensis* differs from the others in that the T-arms of *trnM*, *trnR* and *trnS1* were replaced by a variable loop. *trnP* of *T. malaysiensis* and *trnQ* of *T. kanzawai* still have T-arms while the *trnP*s and *trnQ*s in the other species have lost them. The *trnD* has a only 4 bp well-paired anticodon stem. And the *trnP* of *T. ludeni*, *trnQ* of *T. kanzawai* and *T. phaselus* and *trnV* of *T. malaysiensis* also have one mismatch. Commonly, the 3 bp anticodon of the tRNAs was flanked by 2 bp up-stream and 2 bp down-stream, but *trnI* of *Tetranychus* mitochondria has 3 bp down-stream. These aberrant anticodon loops have been reported for the same tRNA of *P. citri* [13] and *P. ulmi*, which implies that this anticodon loop pattern is universal among *trnI* genes of the Tetranychidae mitochondria. Noncanonical anticodon loop structures are also present in *trnL2* (3 bp down-stream) of *D. pteronyssinus* [16], *trnS2* (3 bp up-stream) of *Camelus bactrianus ferus* [58] and *trnH* (3 bp up-stream) and *trnN* (6 bp down-stream) of *Mesobuthus gibbosus* [59].

### Phylogenetic analysis

**Figure 6:**
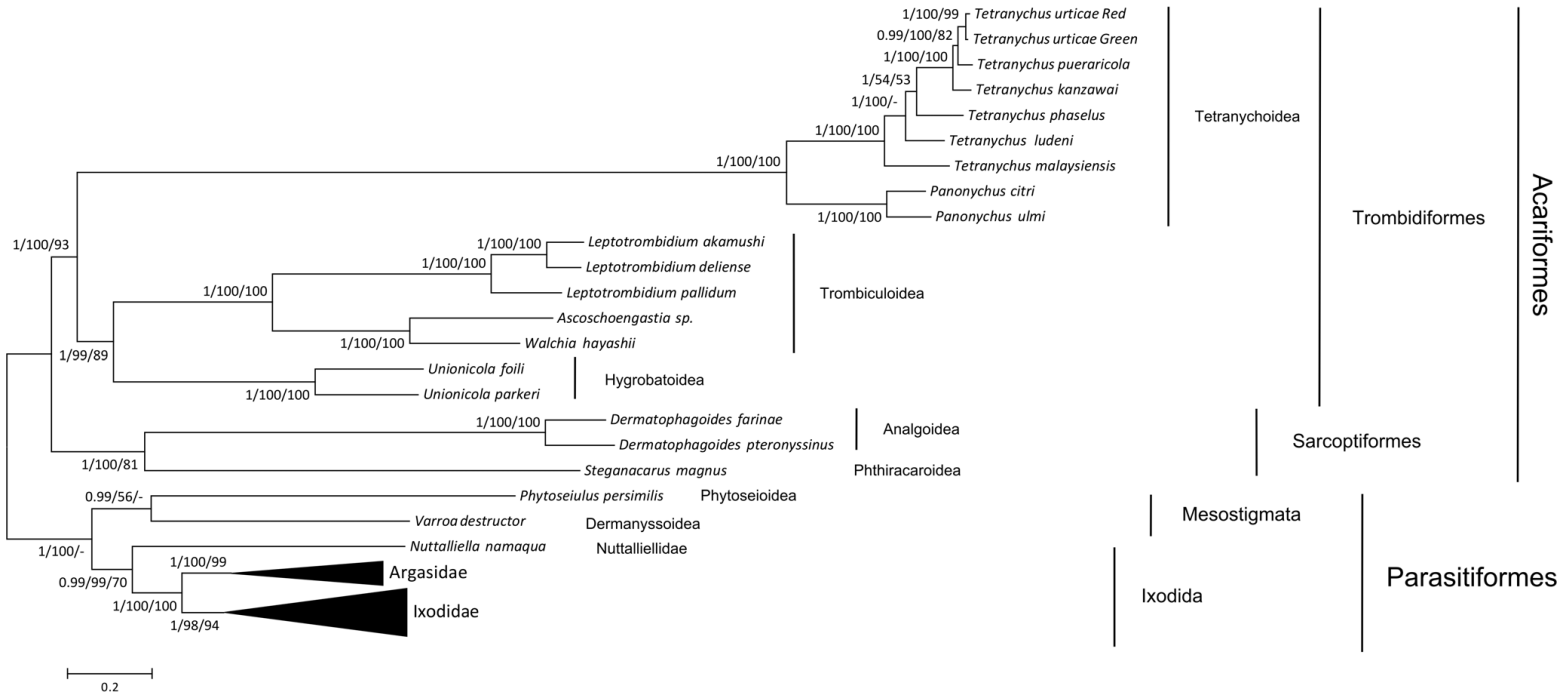
Phylogenetic tree of Acariformes relationships. The tree was inferred from amino acid datasets and rooted with Parasitiformes taxa. Numbers at nodes are percentages from Bayesian posterior probabilities (left), ML bootstrapping (middle) and MP bootstrap support values (right). The nodes that did not support Bayesian inference or had low bootstrap support values were marked as midlines.

Maximum-parsimony (MP), Maximum-Likelihood (ML) and Bayesian inference (BI) phylogenetic trees were constructed based on the concatenated amino acid sequences of the 13 PCGs of the seven *Tetranychus* species and other Acariformes. The three trees had identical topologies (Fig. 6). Almost all nodes were well supported, whereas the split between *T. phaselus* and the other *Tetranychus* species was not supported by the MP tree. The three species of Sarcoptiformes were monophyletic and clustered as a sister group of Trombidiformes, in agreement with a previous analysis [16]. The superfamily Tetranychoidea, which is considered as a member of Trombidiformes, split as a single clade and formed a sister group to other species of Trombidiformes. Another study [16] obtained a similar topology, except that the bootstrap values and BI posterior probabilities for the Tetranychoidea were low. Mitochondrial genomes evolve at high rates. It has been suggested that the three most important factors are rearrangements in the mitochondrial genome, a parasitic lifestyle and small body size [60]. Most species of Acariformes exhibit all three factors and the great variability in branch lengths suggest a high heterogeneous substitution rate among the mt genomes. These problems led to markedly long branches, and the superabundant phylogenetic signals probably led to the distant phylogenetic position of the Tetranychoidea. Identical topologies were constructed by the three approaches with high bootstrap values and high Bayesian posterior probabilities for the position of Tetranychoidea. However, additional mt genome data or trees based on more conserved sequences are needed to improve the phylogeny of Acariformes.

The seven *Tetranychus* species clustered monophyleticly. *T. malaysiensis* was allocated to the basal position of the genus *Tetranychus*. The position of *T. phaselus* was supported by the BI and ML trees, whereas the bootstrapping for MP questioned this node. A phylogenetic tree was constructed based on the mitochondrial genomic sequences of Tetranychoidea (Fig. S6). The topologies of the trees constructed with the three approaches were consistent. Furthermore, a phylogenetic analysis based on nuclear gene sequences of rRNA and ITS supported this analysis (data not shown). However, the position of *T. phaselus* was not supported well by three trees. Some other mitochondrial genomic sequences are needed to improve the phylogenetic analysis for the genus *Tetranychus*. Especially the species has close relationship with *T. phaselus*.

### The green and red forms of *T. urticae*

The green and red forms of *T. urticae* clustered together in the phylogenetic trees and the evolutionary divergence between their mitochondrial genomes was low (0.027±0.002) (Table S6). Pairwise distances among the *Tetranychus* mt genomes except for the value between the two forms of *T. urticae* were 0.089 to 0.211 which is much higher than the distance between the two forms of *T. urticae*. Some slight divergences between two forms are found in the length of 4 bp difference, an AGG codon found in the PCGs of green form, a different start codon in *nad3* gene and a different stop codon in *cox1* gene. However, these limited differences can not be classified as interspecies divergences. Consequently, the close evolutionary distance and limited mitochondrial genome divergence do not support the red forms of *T. urticae* as a new species or subspecies. Between the two forms of *T. urticae*, partial hybrid infertility was discovered [61] and hybrid affinity strongly restricted the gene flow [62]. However, the harboring of *Wolbachia* and *Cardinium* by the two forms [63]–[65] confounds the origin of reproductive abnormality in the hybrid analyses. *Wolbachia* is considered to induce cytoplasmic incompatibility (CI) in *T. urticae*, whereas the levels of CI in different populations varied greatly [66], [67]. Although *Cardinium* did not appear to distort reproduction in *T. urticae* [63], it is still necessary to investigate the CI level between *Wolbachia* and *Cardinium* in *T. urticae*. In conclusion, intracellular bacteria with the ability to manipulate reproduction complicate investigations of the ability of the two forms to interbreed. Further studies are needed to analyze the hybridization in *T. urticae* without the influence of *Wolbachia*, *Cardinium* and other bacteria that can manipulate reproduction.

## Supporting Information

Appendix S1 **Multiple alignments of Acari 13 PCGs' amino acid sequences (13PCGs.meg).** The order of the PCGs is *atp6, atp8, cox1-3, cob, nad1-4, nad4l and nad5–6*. The alignments were performed with ClustalW as implemented in Mega and require the Mega software version 6 to examine it (http://www.megasoftware.net/).
(MEG)

Appendix S2 **Multiple alignments of Tetranychidae mitochondrial genomic sequence (Tetranychidae.meg).** The alignments were performed with ClustalW as implemented in Mega and require the Mega software version 6 to examine it (http://www.megasoftware.net/).
(MEG)

Figure S1 **Species identification by PCR-restriction fragment-length polymorphism.** PCR products were digested by 5 restriction endonucleases (MboII, HinfI, RsaI, DraI, and DdeI). The white arrowheads indicate interspecific variation. M, 100-bp ladder DNA size marker.
(TIF)

Figure S2 **Base composition at each codon position of the 13 PCGs.** Y-axis shows the percentage of each nucleotide.
(TIF)

Figure S3 **Codon usage pattern of each mitochondrial genome.** Numbers to the left refer to the percentage of each codon. Codon families are shown on the X-axis.
(TIF)

Figure S4 **Putative secondary structure of the large-subunit ribosomal RNA of** ***T. urticae*** **.** Inferred Watson-Crick bonds are illustrated by black dots, whereas GU bonds are illustrated by grey dots. The nucleotides with bold text show 100% identity among the seven mitochondrial genomes. The numbering of stem-loops is after [69].
(TIF)

Figure S5 **Putative secondary structure of the small-subunit ribosomal RNA of** ***T. urticae*** **.** Inferred Watson-Crick bonds are illustrated by black dots, whereas GU bonds are illustrated by grey dots. The nucleotides with bold text show 100% identity among the seven mitochondrial genomes. The numbering of stem-loops is after [70].
(TIF)

Figure S6 **Phylogenetic tree of Tetranychoidea relationships.** The tree was inferred from mitochondrial genomic sequences. Numbers at nodes are percentages from Bayesian posterior probabilities (left), ML bootstrapping (middle) and MP bootstrap support values (right).
(TIF)

Table S1 **GenBank accession numbers of mitochondrial genomes for other Acari.**
(DOC)

Table S2 **Date and location of mite collections and GenBank accession numbers of mitochondrial genomes.**
(DOC)

Table S3 **Initial primers and their sequences for PCR amplifications in this study.**
(DOC)

Table S4 **Summary of mitochondrial genome organization of** ***T. urticae*** **(green and red forms),** ***T. kanzawai*** **,** ***T. ludeni*** **,** ***T. malaysiensis*** **,** ***T. phaselus*** **and** ***T. pueraricola*** **.**
(DOC)

Table S5 **Comparison of inferred secondary structures of mitochondrial tRNA genes.**
(DOC)

Table S6 **Pairwise distance between mitochondrial genomes.**
(DOC)
