# Supplementary figures and images for: The Complete Mitochondrial Genomes of Six Species of Tetranychus Provide Insights into the Phylogeny and Evolution of Spider Mites
Source: PLoS One. 2014 Oct 16;9(10):e110625. doi: 10.1371/journal.pone.0110625 (PMC4199730; doi:10.1371/journal.pone.0110625)

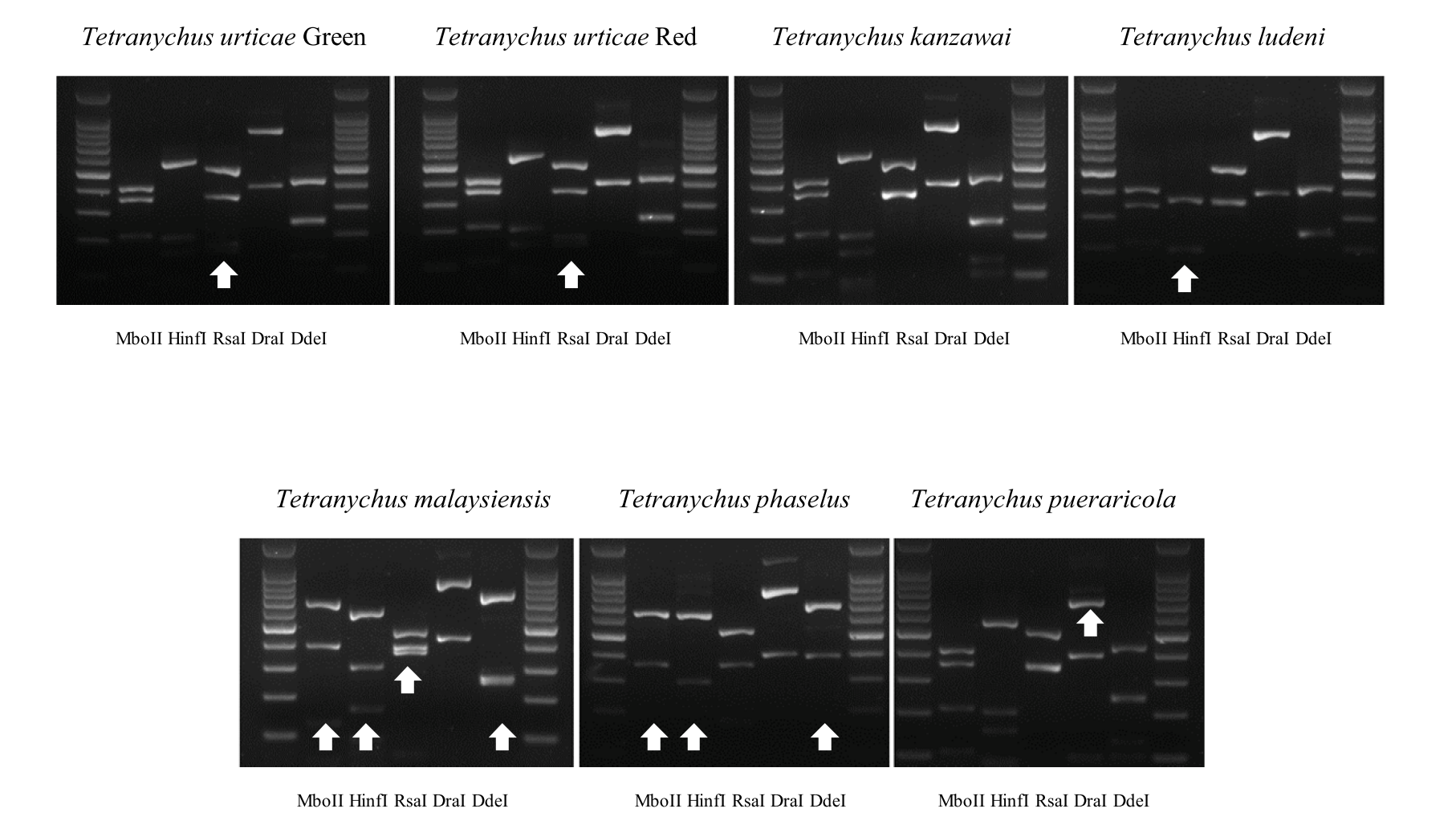

Supplement: Figure S1 — Species identification by PCR-restriction fragment-length polymorphism. PCR products were digested by 5 restriction endonucleases (MboII, HinfI, RsaI, DraI, and DdeI). The white arrowheads indicate interspecific variation. M, 100-bp ladder DNA size marker. (TIF) [file pone.0110625.s003.tif]

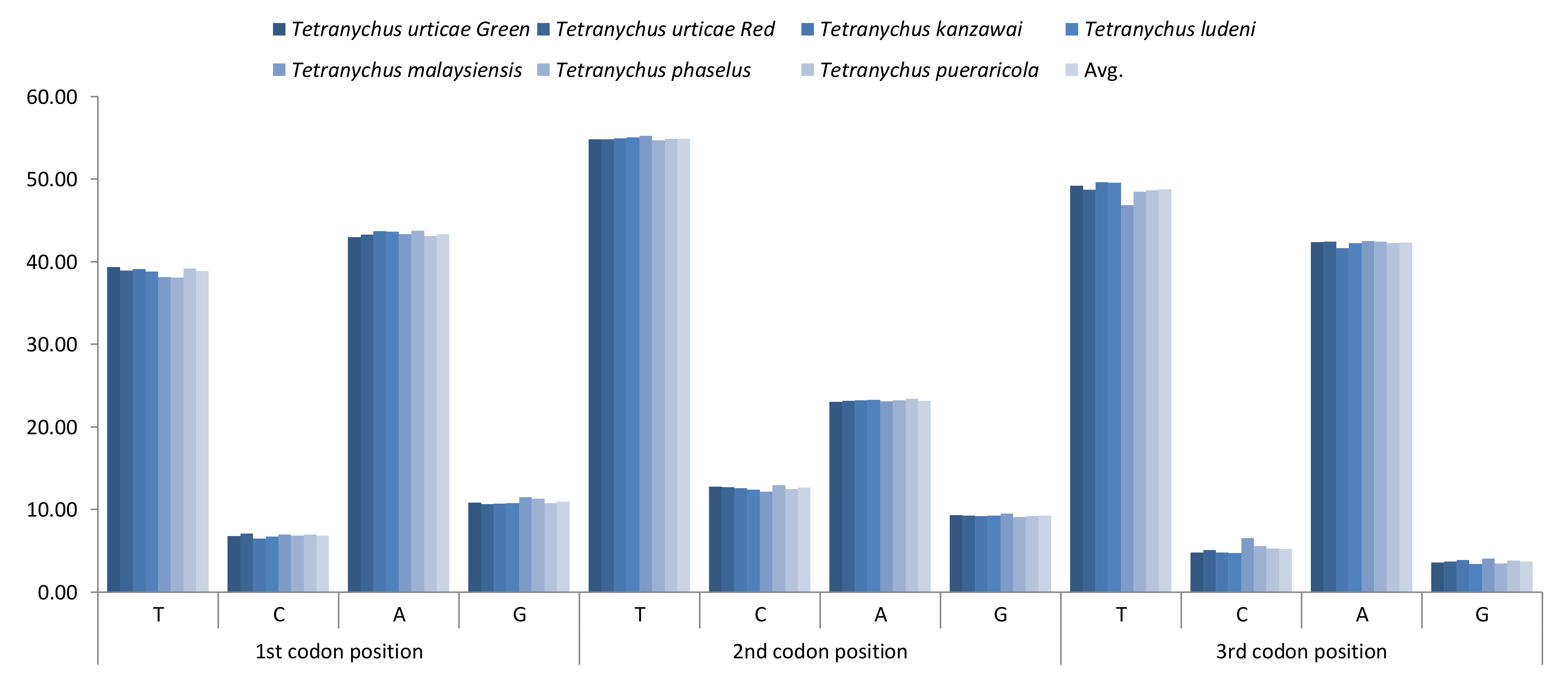

Supplement: Figure S2 — Base composition at each codon position of the 13 PCGs. Y-axis shows the percentage of each nucleotide. (TIF) [file pone.0110625.s004.tif]

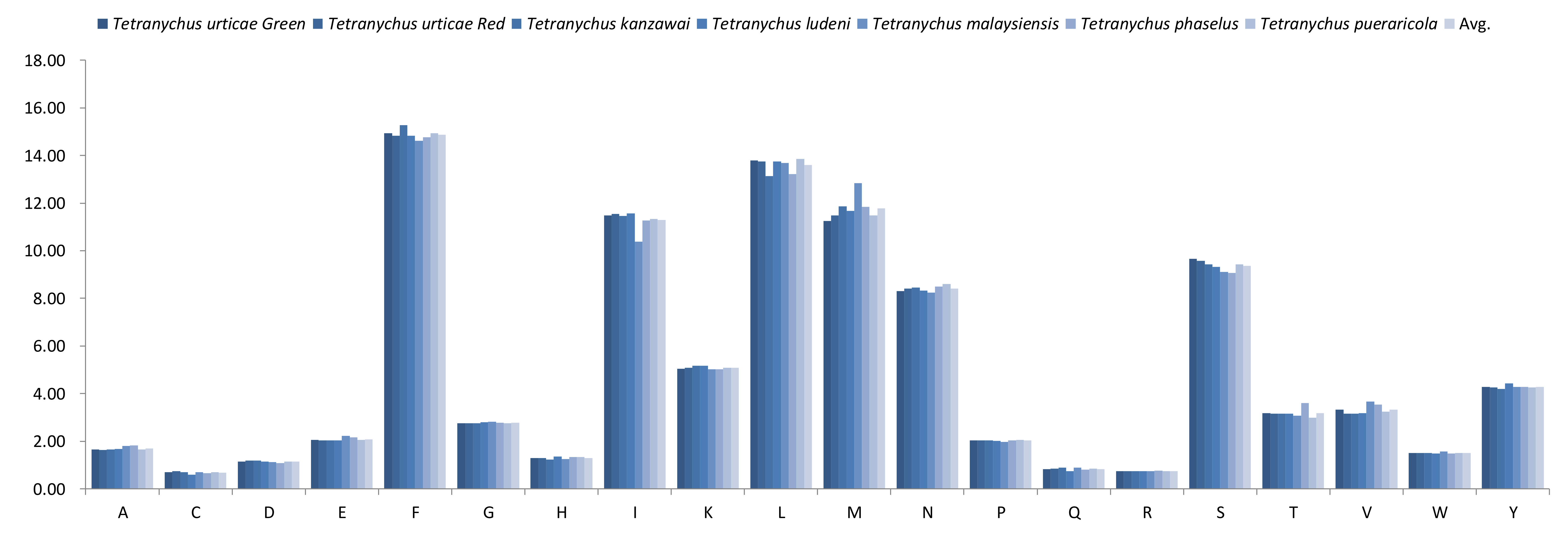

Supplement: Figure S3 — Codon usage pattern of each mitochondrial genome. Numbers to the left refer to the percentage of each codon. Codon families are shown on the X-axis. (TIF) [file pone.0110625.s005.tif]

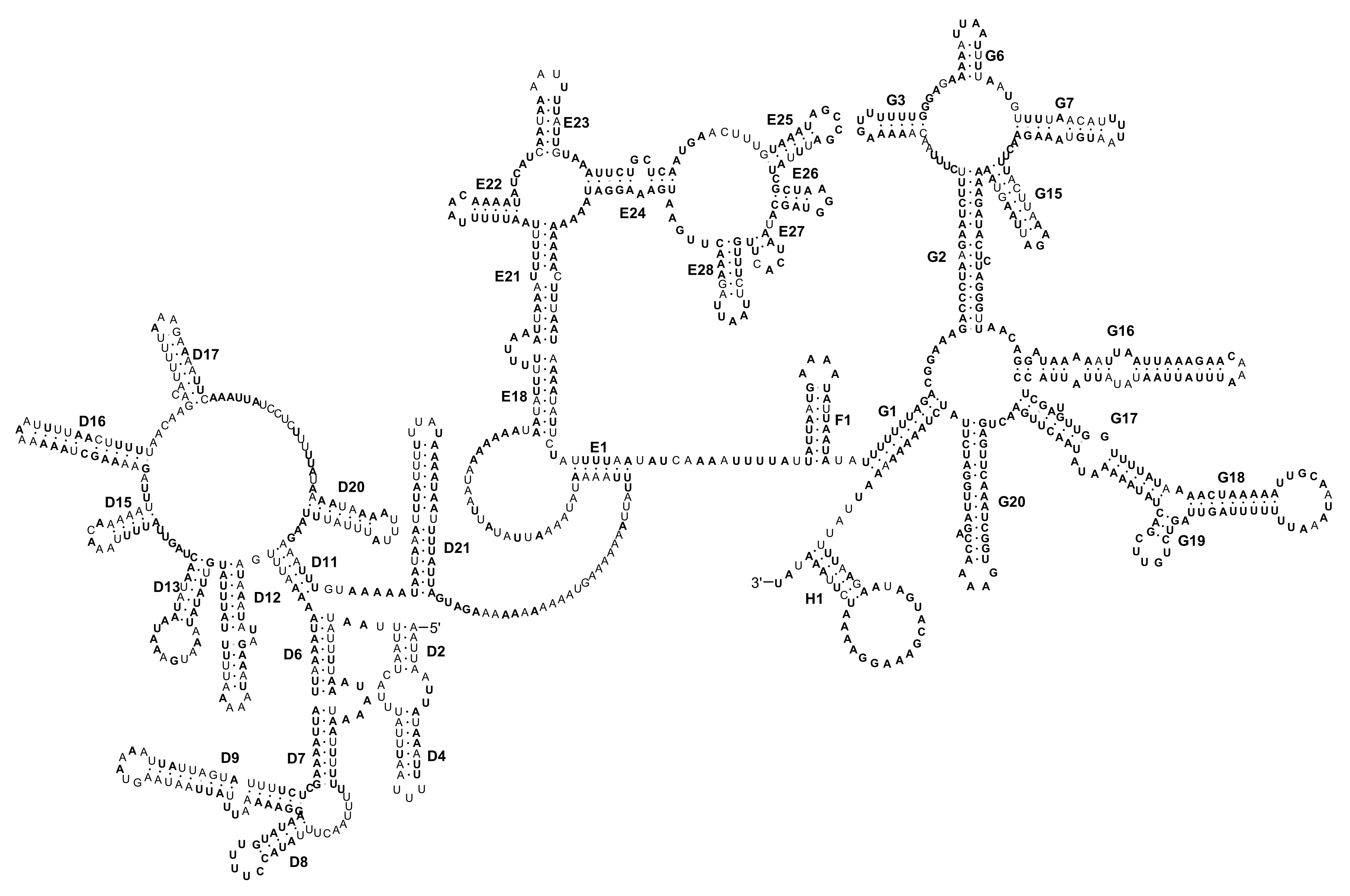

Supplement: Figure S4 — Putative secondary structure of the large-subunit ribosomal RNA of T. urticae . Inferred Watson-Crick bonds are illustrated by black dots, whereas GU bonds are illustrated by grey dots. The nucleotides with bold text show 100% identity among the seven mitochondrial genomes. The numbering of stem-loops is after [69]. (TIF) [file pone.0110625.s006.tif]

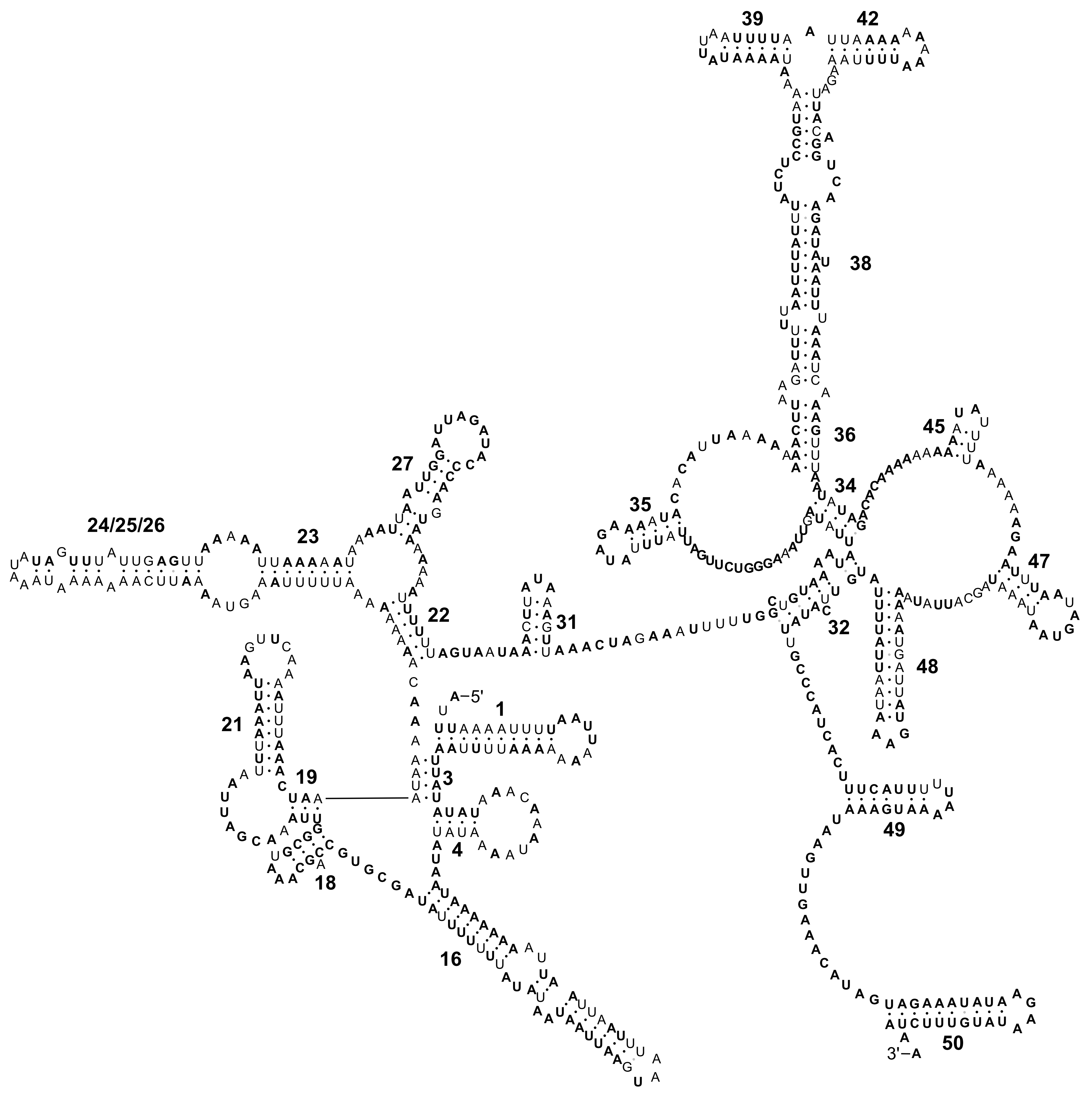

Supplement: Figure S5 — Putative secondary structure of the small-subunit ribosomal RNA of T. urticae . Inferred Watson-Crick bonds are illustrated by black dots, whereas GU bonds are illustrated by grey dots. The nucleotides with bold text show 100% identity among the seven mitochondrial genomes. The numbering of stem-loops is after [70]. (TIF) [file pone.0110625.s007.tif]

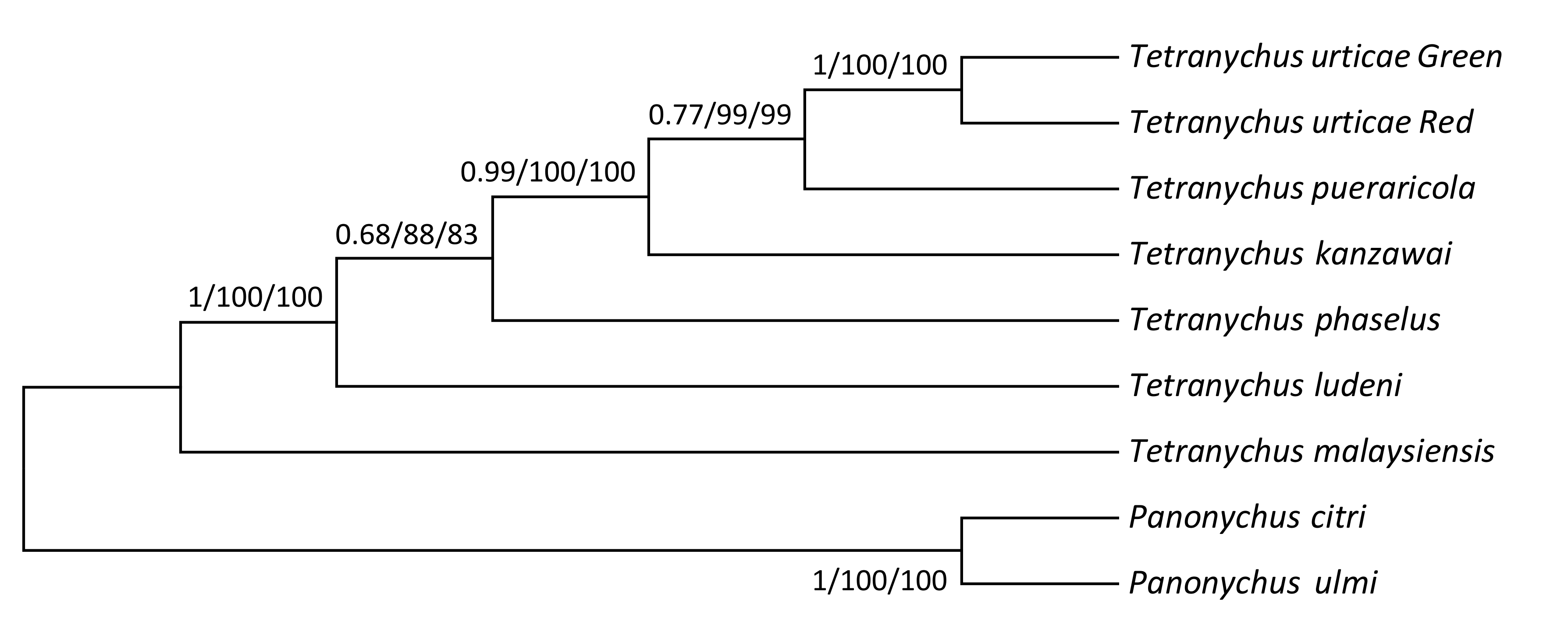

Supplement: Figure S6 — Phylogenetic tree of Tetranychoidea relationships. The tree was inferred from mitochondrial genomic sequences. Numbers at nodes are percentages from Bayesian posterior probabilities (left), ML bootstrapping (middle) and MP bootstrap support values (right). (TIF) [file pone.0110625.s008.tif]
