# Supplementary material for: The Complete Mitochondrial Genomes of Six Species of Tetranychus Provide Insights into the Phylogeny and Evolution of Spider Mites
Source: PLoS One. 2014 Oct 16;9(10):e110625. doi: 10.1371/journal.pone.0110625 (PMC4199730; doi:10.1371/journal.pone.0110625)
Supplement: Table S1 — GenBank accession numbers of mitochondrial genomes for other Acari. (DOC) [file pone.0110625.s009.doc]

## Table S1. GenBank accession numbers of mitochondrial genomes for other Acari

| Species | Classification | GenBank accession number |
| --- | --- | --- |
| *Leptotrombidium pallidum* | Arachnida;Acari;Acariformes | NC_007177 |
| *Leptotrombidium deliense* | Arachnida;Acari;Acariformes | NC_007600 |
| *Leptotrombidium akamushi* | Arachnida;Acari;Acariformes | NC_007601 |
| *Walchia hayashii* | Arachnida;Acari;Acariformes | NC_010595 |
| *Ascoschoengastia sp.* | Arachnida;Acari;Acariformes | NC_010596 |
| *Unionicola foili* | Arachnida;Acari;Acariformes | NC_011036 |
| *Steganacarus magnus* | Arachnida;Acari;Acariformes | NC_011574 |
| *Dermatophagoides pteronyssinus* | Arachnida;Acari;Acariformes | NC_012218 |
| *Panonychus ulmi* | Arachnida;Acari;Acariformes | NC_012571 |
| *Dermatophagoides farinae* | Arachnida;Acari;Acariformes | NC_013184 |
| *Panonychus citri* | Arachnida;Acari;Acariformes | NC_014347 |
| *Unionicola parkeri* | Arachnida;Acari;Acariformes | NC_014683 |
| *Ixodes hexagonus* | Arachnida;Acari;Parasitiformes | NC_002010 |
| *Rhipicephalus sanguineus* | Arachnida;Acari;Parasitiformes | NC_002074 |
| *Ornithodoros moubata* | Arachnida;Acari;Parasitiformes | NC_004357 |
| *Ixodes persulcatus* | Arachnida;Acari;Parasitiformes | NC_004370 |
| *Varroa destructor* | Arachnida;Acari;Parasitiformes | NC_004454 |
| *Carios capensis* | Arachnida;Acari;Parasitiformes | NC_005291 |
| *Haemaphysalis flava* | Arachnida;Acari;Parasitiformes | NC_005292 |
| *Ixodes holocyclus* | Arachnida;Acari;Parasitiformes | NC_005293 |
| *Ornithodoros porcinus* | Arachnida;Acari;Parasitiformes | NC_005820 |
| *Amblyomma triguttatum* | Arachnida;Acari;Parasitiformes | NC_005963 |
| *Ixodes uriae* | Arachnida;Acari;Parasitiformes | NC_006078 |
| *Metaseiulus occidentalis* | Arachnida;Acari;Parasitiformes | NC_009093 |
| *Stylochyrus rarior* | Arachnida;Acari;Parasitiformes | NC_013474 |
| *Phytoseiulus persimilis* | Arachnida;Acari;Parasitiformes | NC_014049 |
| *Amblyomma sphenodonti* | Arachnida;Acari;Parasitiformes | NC_017745 |
| *Bothriocroton concolor* | Arachnida;Acari;Parasitiformes | NC_017756 |
| *Bothriocroton undatum* | Arachnida;Acari;Parasitiformes | NC_017757 |
| *Amblyomma elaphense* | Arachnida;Acari;Parasitiformes | NC_017758 |
| *Aponomma fimbriatum* | Arachnida;Acari;Parasitiformes | NC_017759 |
| *Ixodes ricinus* | Arachnida;Acari;Parasitiformes | NC_018369 |
| *Argas africolumbae* | Arachnida;Acari;Parasitiformes | NC_019642 |
| *Nuttalliella namaqua* | Arachnida;Acari;Parasitiformes | NC_019663 |
| *Amblyomma cajennense* | Arachnida;Acari;Parasitiformes | NC_020333 |
| *Haemaphysalis formosensis* | Arachnida;Acari;Parasitiformes | NC_020334 |
| *Haemaphysalis parva* | Arachnida;Acari;Parasitiformes | NC_020335 |
