# Supplementary material for: The Complete Mitochondrial Genomes of Six Species of Tetranychus Provide Insights into the Phylogeny and Evolution of Spider Mites
Source: PLoS One. 2014 Oct 16;9(10):e110625. doi: 10.1371/journal.pone.0110625 (PMC4199730; doi:10.1371/journal.pone.0110625)
Supplement: Table S2 — Date and location of mite collections and GenBank accession numbers of mitochondrial genomes. (DOC) [file pone.0110625.s010.doc]

**Table S2. Date and location of mite collections and GenBank accession numbers of mitochondrial genomes.**

| Species | Collection Year^1^ | Province | City | Host | GenBank acc. no. | Length (bp) |
| --- | --- | --- | --- | --- | --- | --- |
| *T. kanzawai* | 2013 | Guangdong | Leizhou | Bean | KJ729017 | 13091 |
| *T. ludeni* | 2013 | Guangdong | Shantou | Wax gourd | KJ729018 | 13064 |
| *T. malaysiensis* | 2013 | Hainan | Sanya | Soybean | KJ729019 | 13049 |
| *T. phaselus* | 2009 | Zhejiang | Cixi | Soybean | KJ729020 | 13083 |
| *T. pueraricola* | 2013 | Guangxi | Yongfu | Taro | KJ729021 | 13084 |
| *T. urticae* green | 2009 | Fujian | Quanzhou | Watermelon | KJ729022 | 13096 |
| *T. urticae* red | 2013 | Yunnan | Kunming | Tomato | KJ729023 | 13100 |

^1^All collections were made in July.
