# Supplementary material for: The Complete Mitochondrial Genomes of Six Species of Tetranychus Provide Insights into the Phylogeny and Evolution of Spider Mites
Source: PLoS One. 2014 Oct 16;9(10):e110625. doi: 10.1371/journal.pone.0110625 (PMC4199730; doi:10.1371/journal.pone.0110625)
Supplement: Table S3 — Initial primers and their sequences for PCR amplifications in this study. (DOC) [file pone.0110625.s011.doc]

**Table S3. Initial primers and their sequences for PCR amplifications in this study.**

| Name^a^ | Sequence |
| --- | --- |
| rD02 | GTCGTAACAAGGTTTCCGTAGG |
| HC2 | ATATGCTTAAGTTCAGCGGG |
| T-CO1-F | AAGAGGAGGAGGAGACCCAATT |
| T-CO2-R | AAACCTCTAAAAATAGCGAATACAGC |
| Tu-F | CTCATTTTCATTATGTATTATCAATAGGAGCTGT |
| Tu-R | CAGTAGGGATAGCAATAATTATAGTGGCAGCTGT |
| Tk-F | GCTCACTTTCATTACGTATTATCAATAGGGGCTGT |
| Tk-R | GCTGTGAAGTAAGCTCGAGTATCAACA |
| Tl-F | CTCATTTTCATTATGTATTATCTATAGGAGCTG |
| Tl-R | TGATGCTACAATTCCTGTAAATCCCC |
| Tm-F | CTCATTTTCATTATGTTTTGTCTATAGGAGCTG |
| Tm-R | TGATGCTACAATTCCCGTAAATCCTCC |
| Tph-F | CATTTTCATTATGTTTTATCTATAGGAGCTG |
| Tph-R | AAAACCTAAAAGTCCGATAGATATTATAGCAA |
| Tpu-F | ATTTTACAGCTGCTACTATAATTATTGCTA |
| Tpu-R | AAATCCTAATAAACCGATAGATATTATAGCAA |

^a^ abbreviation of primer name: Tu = *T. urticae*, Tk = *T. kanzawai*, Tl = *T. ludeni*, Tm = *T. malaysiensis*, Tph = *T. phaselus* and Tpu = *T. pueraricola*.
