# Supplementary material for: The Complete Mitochondrial Genomes of Six Species of Tetranychus Provide Insights into the Phylogeny and Evolution of Spider Mites
Source: PLoS One. 2014 Oct 16;9(10):e110625. doi: 10.1371/journal.pone.0110625 (PMC4199730; doi:10.1371/journal.pone.0110625)
Supplement: Table S4 — Summary of mitochondrial genome organization of T. urticae (green and red forms), T. kanzawai , T. ludeni , T. malaysiensis , T. phaselus and T. pueraricola . (DOC) [file pone.0110625.s012.doc]

**Table S4. Summary of mitochondrial genome organization of *T. urticae* (green and red forms), *T. kanzawai*, *T. ludeni*, *T. malaysiensis*, *T. phaselus* and *T. pueraricola*.**

|  |  | *Tetranychus urticae* Green | | | | | *Tetranychus urticae* Red | | | | | *Tetranychus kanzawai* | | | | | *Tetranychus ludeni* | | | | | *Tetranychus malaysiensis* | | | | | *Tetranychus phaselus* | | | | | *Tetranychus pueraricola* | | | | |
| --- | --- | --- | --- | --- | --- | --- | --- | --- | --- | --- | --- | --- | --- | --- | --- | --- | --- | --- | --- | --- | --- | --- | --- | --- | --- | --- | --- | --- | --- | --- | --- | --- | --- | --- | --- | --- |
| Gene | Str^a^ | Position | Size | int^b^ | fcd^c^ | scd^d^ | Position | Size | int | fcd | scd | Position | Size | int | fcd | scd | Position | Size | int | fcd | scd | Position | Size | int | fcd | scd | Position | Size | int | fcd | scd | Position | Size | int | fcd | scd |
| *cox1* | J | 1-1539 | 1539 | 0 | ATA | TAA | 1-1539 | 1539 | 0 | ATA | TAG | 1-1539 | 1539 | 0 | ATA | TAA | 1-1539 | 1539 | 0 | ATA | TAA | 1-1539 | 1539 | 0 | ATC | TAA | 1-1542 | 1542 | 0 | ATT | TAA | 1-1539 | 1539 | 0 | ATT | TAA |
| D-loop | J | 1540-1582 | 43 | 0 |  |  | 1540-1583 | 44 | 0 |  |  | 1540-1583 | 44 | 0 |  |  | 1540-1581 | 42 | 0 |  |  | 1540-1582 | 43 | 0 |  |  | 1543-1583 | 41 | 0 |  |  | 1540-1583 | 44 | 0 |  |  |
| *nad3* | J | 1583-1918 | 336 | 0 | ATA | TAA | 1584-1919 | 336 | 0 | ATG | TAA | 1584-1919 | 336 | 0 | ATA | TAA | 1582-1917 | 336 | 0 | ATT | TAA | 1583-1916 | 334 | 0 | ATT | T | 1584-1919 | 336 | 0 | ATA | TAA | 1584-1919 | 336 | 0 | ATT | TAA |
| *trnN* | J | 1919-1982 | 64 | 0 |  |  | 1920-1984 | 65 | 0 |  |  | 1920-1982 | 63 | 0 |  |  | 1918-1977 | 60 | 0 |  |  | 1917-1977 | 61 | 0 |  |  | 1920-1983 | 64 | 0 |  |  | 1920-1982 | 63 | 0 |  |  |
| *trnD* | N | 1983-2036 | 54 | 0 |  |  | 1985-2037 | 53 | 0 |  |  | 1983-2036 | 54 | 0 |  |  | 1978-2034 | 57 | 0 |  |  | 1978-2034 | 57 | 0 |  |  | 1984-2037 | 54 | 0 |  |  | 1983-2035 | 53 | 0 |  |  |
| *trnL1* | J | 2036-2089 | 54 | -1 |  |  | 2037-2090 | 54 | -1 |  |  | 2035-2088 | 54 | -2 |  |  | 2034-2088 | 55 | -1 |  |  | 2033-2087 | 55 | -2 |  |  | 2037-2091 | 55 | -1 |  |  | 2034-2089 | 56 | -2 |  |  |
| *trnE* | N | 2089-2139 | 51 | -1 |  |  | 2090-2140 | 51 | -1 |  |  | 2088-2136 | 49 | -1 |  |  | 2088-2138 | 51 | -1 |  |  | 2087-2135 | 49 | -1 |  |  | 2091-2142 | 52 | -1 |  |  | 2088-2137 | 50 | -2 |  |  |
| *rrnL* | J | 2140-3138 | 998 | 0 |  |  | 2141-3139 | 998 | 0 |  |  | 2137-3135 | 998 | 0 |  |  | 2139-3121 | 982 | 0 |  |  | 2136-3118 | 983 | 0 |  |  | 2143-3136 | 993 | 0 |  |  | 2138-3129 | 992 | 0 |  |  |
| *trnR* | J | 3139-3189 | 51 | 0 |  |  | 3140-3190 | 51 | 0 |  |  | 3136-3185 | 50 | 0 |  |  | 3122-3170 | 49 | 0 |  |  | 3119-3169 | 51 | 0 |  |  | 3137-3187 | 51 | 0 |  |  | 3130-3179 | 50 | 0 |  |  |
| *nad4L* | N | 3196-3441 | 246 | 6 | ATT | TAA | 3198-3443 | 246 | 7 | ATT | TAA | 3191-3436 | 246 | 5 | ATA | TAA | 3177-3422 | 246 | 6 | ATA | TAG | 3171-3416 | 246 | 1 | ATA | TAG | 3190-3435 | 246 | 2 | ATC | TAA | 3184-3429 | 246 | 3 | ATT | TAA |
| *trnP* | N | 3442-3485 | 43 | 0 |  |  | 3444-3487 | 44 | 0 |  |  | 3437-3480 | 44 | 0 |  |  | 3423-3465 | 43 | 0 |  |  | 3417-3461 | 45 | 0 |  |  | 3436-3478 | 43 | 0 |  |  | 3430-3473 | 44 | 0 |  |  |
| *trnF* | N | 3486-3544 | 59 | 0 |  |  | 3488-3546 | 59 | 0 |  |  | 3481-3539 | 59 | 0 |  |  | 3466-3527 | 62 | 0 |  |  | 3462-3521 | 60 | 0 |  |  | 3479-3535 | 57 | 0 |  |  | 3474-3532 | 59 | 0 |  |  |
| *cox3* | N | 3545-4331 | 787 | 0 | ATG | T | 3547-4333 | 787 | 0 | ATG | T | 3540-4326 | 787 | 0 | ATG | T | 3528-4314 | 786 | 0 | ATG | T | 3522-4305 | 784 | 0 | ATG | T | 3536-4322 | 787 | 0 | ATG | T | 3533-4319 | 787 | 0 | ATG | T |
| *atp6* | N | 4332-4950 | 619 | 0 | ATG | T | 4334-4952 | 619 | 0 | ATG | T | 4327-4945 | 619 | 0 | ATG | T | 4315-4933 | 619 | 0 | ATG | T | 4306-4924 | 619 | 0 | ATG | TAG | 4323-4941 | 619 | 0 | ATG | T | 4320-4938 | 619 | 0 | ATG | T |
| *atp8* | N | 4951-5080 | 130 | 0 | ATA | T | 4953-5082 | 130 | 0 | ATA | T | 4946-5075 | 130 | 0 | ATA | T | 4934-5063 | 130 | 0 | ATT | T | 4925-5054 | 130 | 0 | TTG | T | 4942-5071 | 130 | 0 | ATT | T | 4939-5068 | 130 | 0 | ATT | T |
| *trnK* | N | 5081-5142 | 62 | 0 |  |  | 5083-5144 | 62 | 0 |  |  | 5076-5138 | 63 | 0 |  |  | 5064-5125 | 62 | 0 |  |  | 5055-5116 | 62 | 0 |  |  | 5072-5133 | 62 | 0 |  |  | 5069-5130 | 62 | 0 |  |  |
| *cox2* | N | 5148-5786 | 639 | 5 | ATG | TAA | 5150-5788 | 639 | 5 | ATG | TAA | 5144-5782 | 639 | 5 | ATG | TAA | 5133-5771 | 639 | 7 | ATG | TAA | 5120-5759 | 640 | 3 | ATG | T | 5141-5779 | 639 | 7 | ATG | TAA | 5136-5774 | 639 | 5 | ATG | TAG |
| *trnY* | N | 5798-5844 | 47 | 11 |  |  | 5802-5848 | 47 | 13 |  |  | 5790-5836 | 47 | 7 |  |  | 5779-5829 | 51 | 8 |  |  | 5764-5812 | 49 | 4 |  |  | 5785-5833 | 49 | 5 |  |  | 5782-5828 | 47 | 7 |  |  |
| *rrnS* | J | 5845-6484 | 640 | 0 |  |  | 5849-6491 | 642 | 0 |  |  | 5837-6479 | 643 | 0 |  |  | 5830-6458 | 629 | 0 |  |  | 5813-6442 | 630 | 0 |  |  | 5834-6462 | 629 | 0 |  |  | 5829-6473 | 645 | 0 |  |  |
| *trnG* | J | 6485-6539 | 55 | 0 |  |  | 6492-6546 | 55 | 0 |  |  | 6480-6534 | 55 | 0 |  |  | 6459-6515 | 57 | 0 |  |  | 6443-6497 | 55 | 0 |  |  | 6463-6518 | 56 | 0 |  |  | 6474-6528 | 55 | 0 |  |  |
| *trnT* | N | 6540-6593 | 54 | 0 |  |  | 6547-6600 | 54 | 0 |  |  | 6535-6588 | 54 | 0 |  |  | 6520-6575 | 56 | 4 |  |  | 6505-6560 | 56 | 7 |  |  | 6520-6573 | 54 | 1 |  |  | 6529-6582 | 54 | 0 |  |  |
| *nad1* | N | 6596-7451 | 856 | 2 | ATT | T | 6603-7458 | 856 | 2 | ATT | T | 6591-7446 | 856 | 2 | ATT | T | 6578-7433 | 856 | 2 | ATT | T | 6563-7415 | 853 | 2 | ATA | T | 6577-7432 | 856 | 3 | ATT | T | 6585-7440 | 856 | 2 | ATT | T |
| *trnL2* | N | 7452-7513 | 62 | 0 |  |  | 7459-7520 | 62 | 0 |  |  | 7447-7508 | 62 | 0 |  |  | 7434-7495 | 62 | 0 |  |  | 7416-7477 | 62 | 0 |  |  | 7432-7490 | 59 | -1 |  |  | 7441-7504 | 64 | 0 |  |  |
| *trnQ* | N | 7506-7552 | 47 | -8 |  |  | 7513-7559 | 46 | -8 |  |  | 7501-7548 | 48 | -8 |  |  | 7488-7533 | 46 | -8 |  |  | 7470-7516 | 47 | -8 |  |  | 7483-7529 | 47 | -8 |  |  | 7497-7543 | 47 | -8 |  |  |
| *trnC* | J | 7553-7602 | 50 | 0 |  |  | 7560-7608 | 48 | 0 |  |  | 7549-7597 | 49 | 0 |  |  | 7534-7585 | 52 | 0 |  |  | 7517-7567 | 51 | 0 |  |  | 7530-7580 | 51 | 0 |  |  | 7544-7593 | 50 | 0 |  |  |
| *cob* | J | 7614-8678 | 1065 | 11 | ATT | TAA | 7620-8684 | 1065 | 11 | ATT | TAA | 7607-8671 | 1065 | 9 | ATT | TAA | 7598-8662 | 1065 | 12 | ATA | TAA | 7576-8640 | 1065 | 8 | ATT | TAA | 7589-8653 | 1065 | 8 | ATT | TAA | 7603-8667 | 1065 | 9 | ATT | TAA |
| *trnS2* | N | 8677-8720 | 44 | -2 |  |  | 8684-8726 | 43 | -1 |  |  | 8671-8714 | 44 | -1 |  |  | 8662-8704 | 43 | -1 |  |  | 8640-8683 | 44 | -1 |  |  | 8654-8696 | 43 | 0 |  |  | 8667-8710 | 44 | -1 |  |  |
| *trnA* | N | 8716-8763 | 48 | -5 |  |  | 8722-8769 | 48 | -5 |  |  | 8710-8760 | 51 | -5 |  |  | 8700-8744 | 45 | -5 |  |  | 8679-8729 | 51 | -5 |  |  | 8692-8739 | 48 | -5 |  |  | 8706-8755 | 50 | -5 |  |  |
| *nad6* | J | 8764-9163 | 400 | 0 | ATA | T | 8770-9169 | 400 | 0 | ATA | T | 8761-9160 | 400 | 0 | ATA | T | 8745-9144 | 400 | 0 | ATA | T | 8730-9126 | 397 | 0 | ATC | T | 8741-9140 | 400 | 1 | ATA | T | 8756-9155 | 400 | 0 | ATA | T |
| *nad4* | J | 9164-10318 | 1155 | 0 | ATG | TAA | 9170-10324 | 1155 | 0 | ATG | TAA | 9161-10315 | 1155 | 0 | ATG | TAA | 9145-10298 | 1154 | 0 | ATG | TA | 9127-10281 | 1155 | 0 | ATC | TAA | 9141-10295 | 1155 | 0 | ATG | TAA | 9156-10310 | 1155 | 0 | ATG | TAA |
| *trnH* | J | 10318-10373 | 56 | -1 |  |  | 10324-10379 | 56 | -1 |  |  | 10315-10369 | 55 | -1 |  |  | 10298-10352 | 55 | -1 |  |  | 10281-10335 | 55 | -1 |  |  | 10300-10355 | 56 | 4 |  |  | 10310-10364 | 55 | -1 |  |  |
| *nad5* | J | 10374-11939 | 1566 | 0 | ATT | TAA | 10380-11945 | 1566 | 0 | ATT | TAA | 10370-11935 | 1566 | 0 | ATT | TAA | 10353-11918 | 1566 | 0 | ATA | TAA | 10336-11895 | 1560 | 0 | ATT | TAA | 10356-11921 | 1566 | 0 | ATA | TAA | 10365-11930 | 1566 | 0 | ATT | TAG |
| *trnW* | J | 11949-12003 | 55 | 9 |  |  | 11955-12009 | 55 | 9 |  |  | 11946-12000 | 55 | 10 |  |  | 11920-11974 | 55 | 1 |  |  | 11905-11959 | 55 | 9 |  |  | 11935-11988 | 54 | 13 |  |  | 11940-11994 | 55 | 9 |  |  |
| *nad2* | J | 12004-12892 | 889 | 0 | ATA | T | 12010-12898 | 889 | 0 | ATA | T | 12001-12889 | 889 | 0 | ATA | T | 11975-12863 | 889 | 0 | ATA | T | 11960-12848 | 889 | 0 | ATA | T | 11990-12878 | 889 | 1 | ATA | T | 11995-12883 | 889 | 0 | ATA | T |
| *trnM* | J | 12893-12947 | 55 | 0 |  |  | 12899-12953 | 55 | 0 |  |  | 12890-12944 | 55 | 0 |  |  | 12864-12919 | 56 | 0 |  |  | 12849-12906 | 58 | 0 |  |  | 12878-12933 | 56 | -1 |  |  | 12884-12938 | 55 | 0 |  |  |
| *trnS1* | J | 12953-12998 | 46 | 5 |  |  | 12959-13004 | 46 | 5 |  |  | 12951-12996 | 46 | 6 |  |  | 12924-12969 | 46 | 4 |  |  | 12910-12954 | 45 | 3 |  |  | 12939-12984 | 46 | 5 |  |  | 12945-12990 | 46 | 6 |  |  |
| *trnV* | J | 13002-13049 | 48 | 3 |  |  | 13008-13055 | 48 | 3 |  |  | 12999-13046 | 48 | 2 |  |  | 12971-13017 | 47 | 1 |  |  | 12956-13001 | 46 | 1 |  |  | 12986-13032 | 47 | 1 |  |  | 12994-13040 | 47 | 3 |  |  |
| *trnI* | J | 13054-13096 | 43 | 4 |  |  | 13058-13100 | 43 | 4 |  |  | 13048-13091 | 44 | 1 |  |  | 13022-13064 | 43 | 4 |  |  | 13006-13049 | 44 | 4 |  |  | 13040-13083 | 44 | 7 |  |  | 13042-13084 | 43 | 1 |  |  |

^a^ str = strand; genes coded in N- or J- strand are symbolized with N or J. ^b^ int = intergenic nucleotides, positive values indicate gap nucleotides and negative values indicate overlapped nucleotides between two adjacent genes. ^c^ fcd = first codon. ^d^ scd = stop codon.
