# Supplementary material for: The Complete Mitochondrial Genomes of Six Species of Tetranychus Provide Insights into the Phylogeny and Evolution of Spider Mites
Source: PLoS One. 2014 Oct 16;9(10):e110625. doi: 10.1371/journal.pone.0110625 (PMC4199730; doi:10.1371/journal.pone.0110625)
Supplement: Table S5 — Comparison of inferred secondary structures of mitochondrial tRNA genes. (DOC) [file pone.0110625.s013.doc]

**Table S5. Comparison of inferred secondary structures of mitochondrial tRNA genes.**

| Gene | Species | AA-arm |  | D-arm |  | AC-arm | V-loop | T-arm | AA-arm |
| --- | --- | --- | --- | --- | --- | --- | --- | --- | --- |
| *trnN* | *Tetranychus kanzawai* | UUAAAAG | AA | GCUUAAAGC | A | UUUUGUU**GUU**AACAAAA | AGUA | GAAAAAUAUUUUC | CUUUUAAA |
|  | *Tetranychus ludeni* | UUAAAAG | AA | GCUUUAAGC | A | UUUUGUU**GUU**AACAAAA | AGUA | GAAAAUUUUC | CUUUUAAA |
|  | *Tetranychus malaysiensis* | UUAAAAG | AA | GCUUUAAGC | G | UUUUGUU**GUU**AACAAAA | AGUA | GAAAAAAAUUUC | CUUUUAAA |
|  | *Tetranychus phaselus* | UUAAAAG | AA | GCUUAAAGC | A | UUUUGUU**GUU**AACAAAA | AGAA | GAAAAAAUAUUUUC | CUUUUAAA |
|  | *Tetranychus pueraricola* | UUAAAAG | AA | GCUUAAAGC | A | UUUUGUU**GUU**AACAAAA | AGUA | GAAAAUAAUUUUC | CUUUUAAA |
|  | *Tetranychus urticae* Green | UUAAAAG | AA | GCUUAAAGC | A | UUUUGUU**GUU**AACAAAA | AGUA | GAAAAUAUUUUUUC | CUUUUAAA |
|  | *Tetranychus urticae* Red | UUAAAAG | AA | GCUUAAAGC | A | UUUUGUU**GUU**AACAAAA | AGUA | GAAAAUAUUUUUUUC | CUUUUAAA |
| *trnD* | *Tetranychus kanzawai* | AAGAUAA | UAUGAAU |  |  | UAAAUU**GUC**AAUUUA | UUUUUU | CUUUUUAGU | UUAUCUUA |
|  | *Tetranychus ludeni* | AAGAUAA | UUUUGUU |  |  | UAAAUU**GUC**AAUUUA | UAUUUU | CUUUAAAAGU | UUAUCUUA |
|  | *Tetranychus malaysiensis* | AAGAUAA | GUGUAUU |  |  | UAAAUU**GUC**AAUUUA | UUUUU | UCUUUUGGA | UUAUCUUA |
|  | *Tetranychus phaselus* | AAGAUAA | UUAUAUU |  |  | UAAAUU**GUC**AAUUUA | UUUUUU | CUUUUAGU | UUAUCUUA |
|  | *Tetranychus pueraricola* | AAGAUAA | UUUUAUU |  |  | UAAAUU**GUC**AAUUUA | UUUUUU | CUUUUAGU | UUAUCUUA |
|  | *Tetranychus urticae* Green | AAGAUAA | UGUCAUU |  |  | UAAAUU**GUC**AAUUUA | UUUUUU | CUUUAAGU | UUAUCUUA |
|  | *Tetranychus urticae* Red | AAGAUAA | UUUCAUU |  |  | UAAAUU**GUC**AAUUUA | UUUUUU | CUUUAAGU | UUAUCUUA |
| *trnL1* | *Tetranychus kanzawai* | UUUCAUU | GGAGU | AUUAAAUAU | A | UUAAGUU**UAG**AACUUAA | AGAUUAA |  | AAUGAAAA |
|  | *Tetranychus ludeni* | UUUCAUU | AAAGU | AUAAAAAUAU | A | UUAAGUU**UAG**AACUUAA | AGAAAAA |  | AAUGAAAA |
|  | *Tetranychus malaysiensis* | UUUCAUU | AAAGU | AUAUAAAUAC | A | UUAGGUU**UAG**AACUUAA | AGAAAAA |  | AAUGAAAA |
|  | *Tetranychus phaselus* | UUUCAUU | AAAGU | AUUAAAAUAU | A | UUAAGUU**UAG**AACUUAA | AGAAAAG |  | AAUGAAAA |
|  | *Tetranychus pueraricola* | UUUCAUU | AAAGU | AUAAAAAUAU | A | UUAAGUU**UAG**AACUUAA | AGAUUAAA |  | AAUGAAAA |
|  | *Tetranychus urticae* Green | UUUCAUU | AAAGU | AUAAAAUAU | A | UUAAGUU**UAG**AACUUAA | AGGUUAA |  | AAUGAAAA |
|  | *Tetranychus urticae* Red | UUUCAUU | AAAGU | AUUAAAUAU | A | UUAAGUU**UAG**AACUUAA | AGAUUAA |  | AAUGAAAU |
| *trnE* | *Tetranychus kanzawai* | AUUCUGU | UUUG |  |  | AAAGAUU**UUC**AUUCUUU | CAAA | AAUAAAAUU | ACAGAAUA |
|  | *Tetranychus ludeni* | AUUCUGU | UUCA |  |  | AAAGAUU**UUC**ACUCUUU | UUUUAUC | AAAAAUU | ACAGAAUU |
|  | *Tetranychus malaysiensis* | AUUCUGU | UGUA |  |  | AAAGAUU**UUC**ACUCUUU | AAUUUUU | AGUUU | ACAGAAUU |
|  | *Tetranychus phaselus* | AUUCUGU | UAAA |  |  | AAAGAUU**UUC**AUUCUUU | UUAUU | UAUUGUAUA | ACAGAAUU |
|  | *Tetranychus pueraricola* | AUUCUGU | UUAA |  |  | AAAGAUU**UUC**AUUCUUU | UUAA | AAUUAAAUU | ACAGAAUU |
|  | *Tetranychus urticae* Green | AUUCUGU | UAAA |  |  | AAAGAUU**UUC**AUUCUUU | UUAA | AAUUAAAUU | ACAGAAUU |
|  | *Tetranychus urticae* Red | AUUCUGU | UAAA |  |  | AAAGAUU**UUC**AUUCUUU | UAAA | AAUUAAAUU | ACAGAAUU |
| *trnR* | *Tetranychus kanzawai* | UUUUGAU | AAA | UAAAAA | U | UUUGAUU**UCG**AAUCAAA | AAA | UAUAA | AUCAAUAA |
|  | *Tetranychus ludeni* | AAUUGAU | UAA | UAAUA | U | UUUGAUU**UCG**AAUCAAA | AAA | UAAAA | AUCAAAUA |
|  | *Tetranychus malaysiensis* | UUUUGAU | AC | AUAAAAU | U | UUUGAUU**UCG**AAUCAAA | AAAAGAUAA |  | AUCAAAAG |
|  | *Tetranychus phaselus* | AAUUGAU | AAA | UAAAAG | U | UUUGAUU**UCG**AAUCAAA | AAA | UUAAUA | AUCAAAAA |
|  | *Tetranychus pueraricola* | AUUUGAU | AAA | UAAAA | U | UUUGAUU**UCG**AAUCAAA | AA | UGAAUUA | AUCAAUAA |
|  | *Tetranychus urticae* Green | ACUUGAU | AAA | UAAAAG | U | UUUGAUU**UCG**AAUCAAA | AG | UAUACUA | AUCAAUAA |
|  | *Tetranychus urticae* Red | AUUUGAU | AAA | UAAAAG | U | UUUGAUU**UCG**AAUCAAA | AG | UAUACUA | AUCAAUAA |
| *trnP* | *Tetranychus kanzawai* | CAGAU | UUAAUUUAU |  |  | AUUAAUU**UGG**AUUUAAU | AUUUUUU |  | AUCUGA |
|  | *Tetranychus ludeni* | CAGAUU | UCAAUU |  |  | UUAAUU**UGG**AUUUAA | UUUUUUUUU |  | AAUCUGA |
|  | *Tetranychus malaysiensis* | CAGAUU | AAAGA |  |  | AUUAAUU**UGG**AUUUAGU | UU | UUAAAAAA | AAUCUGA |
|  | *Tetranychus phaselus* | CAGAUU | UAUCU |  |  | AUUAAUU**UGG**AUUUAAU | UAUUUUUU |  | AAUCUGA |
|  | *Tetranychus pueraricola* | CAGAU | UUUACUUAU |  |  | AUUAAUU**UGG**AUUUAAU | AUUUUUU |  | AUCUGA |
|  | *Tetranychus urticae* Green | CAGAU | UUAAUUUGU |  |  | AUUAAUU**UGG**AUUUAAU | AUUUUUU |  | AUCUGA |
|  | *Tetranychus urticae* Red | CAGAU | UUAAUUUGU |  |  | AUUAAUU**UGG**AUUUAAU | AUUUUUU |  | AUCUGA |
| *trnF* | *Tetranychus kanzawai* | AUUUUAA | UAAU | UAAACUCUUUAAAAUA | A | UUACAUU**GAA**GAUGUAA | UUUUAA |  | UUGGGGUA |
|  | *Tetranychus ludeni* | AUUUUAA | UAUUUAAU | UAAACUCUUAAUUUA | U | UUACAUU**GAA**GAUGUAA | AACUUA |  | UUGGAGUA |
|  | *Tetranychus malaysiensis* | AUUUUAA | UGUA | UAAACUCUUUAAAAAA | A | UUACAUU**GAA**GAUGUAA | UAUUUUG |  | UUGGAGUA |
|  | *Tetranychus phaselus* | AUUUUAA | UGUU | UAAACUCUUUUUUA | U | UUACAUU**GAA**GAUGUAA | UUUUUA |  | UUAGAGUA |
|  | *Tetranychus pueraricola* | AUUUUAA | UAAUU | AAACUCUUUAAAGUU | U | UUACAUU**GAA**GAUGUAA | AUUUAA |  | UUGGAGUA |
|  | *Tetranychus urticae* Green | AUUUUAA | UAAU | UAAACUCUUUAAAGUA | U | UUACAUU**GAA**GAUGUAA | UUUAAA |  | UUGGAGUA |
|  | *Tetranychus urticae* Red | AUUUUAA | UAAU | UAAACUCUUUAAAAUA | U | UUACAUU**GAA**GAUGUAA | UUUUAA |  | UUGGAGUA |
| *trnK* | *Tetranychus kanzawai* | CAUCAAA | UG | GCUGAGUUAAGC | A | UCAACUU**CUU**AAGUUGA | AUAC | AGAAUUUUUUCU | UUUGAUGA |
|  | *Tetranychus ludeni* | CGUCAAA | UG | GCUGAUUUAAGC | G | AUAACUU**CUU**AAGUUAU | UUAU | AGGUUAAACCU | UUUGAUGA |
|  | *Tetranychus malaysiensis* | CGUCAAA | UG | GCUGAUUAAAGC | G | AUAACUU**CUU**AAGUUAU | UUAU | AGAAUUUUUCU | UUUGAUGA |
|  | *Tetranychus phaselus* | CAUCAAA | UG | GCUGAAUUAAAGC | G | GUAACUU**CUU**AAGUUAU | UUAU | AGAUUAAAUCU | UUUGAUGA |
|  | *Tetranychus pueraricola* | CAUCAAA | UG | GCUGAAUAAAGC | G | UUAACUU**CUU**AAGUUAA | UUAU | AGAUUAAAUCU | UUUGAUGA |
|  | *Tetranychus urticae* Green | CAUCAAA | UG | GCUGAAUAAAGC | A | UUAACUU**CUU**AAGUUAA | UUAU | AGAUAUAAUCU | UUUGAUGA |
|  | *Tetranychus urticae* Red | CAUCAAA | UG | GCUGAUUAAAGC | A | UUAACUU**CUU**AAGUUAA | UUAU | AGAUAAAAUCU | UUUGAUGA |
| *trnY* | *Tetranychus kanzawai* | UUUUGAC | UU | UAAAA | A | AUUUAUU**GUA**AAUAAAU | UUUAAUU |  | GUCAAUUU |
|  | *Tetranychus ludeni* | UUUUGAU | UUU | UUAUUAA | A | AUUUAUU**GUA**AAUAAAU | UUUUAAUU |  | AUCAAAUU |
|  | *Tetranychus malaysiensis* | UUUUGAU | UUU | UUUGUG | A | AUUUAUU**GUA**AAUAAAU | UUUUAUU |  | AUCAAAUA |
|  | *Tetranychus phaselus* | UUUUGAC | UU | UAAUA | UA | AUUUAUU**GUA**AAUAAAU | UUUUUAUU |  | GUCAAAUG |
|  | *Tetranychus pueraricola* | UUUUGAC | UU | UAAUA | A | AUUUAUU**GUA**AAUAAAU | UUUAAUU |  | GUCAAUUU |
|  | *Tetranychus urticae* Green | UUUUGAC | UU | UAAAA | A | AUUUAUU**GUA**AAUAAAU | UUUAAUU |  | GUCAAUUU |
|  | *Tetranychus urticae* Red | UUUUGAC | UU | UAAAA | A | AUUUAUU**GUA**AAUAAAU | UUUAAUU |  | GUCAAUUU |
| *trnG* | *Tetranychus kanzawai* | AUUUUCU | AA | AUACCCCCCGUAU | A | UUCAAUU**UCC**AAUUGAA | AAGAAUA |  | AGAAAAUA |
|  | *Tetranychus ludeni* | AUUUUCU | AA | AUACCCCCCCCGUAU | A | UUCAAUU**UCC**AAUUGAA | AAGUAAA |  | AGAAAAUA |
|  | *Tetranychus malaysiensis* | AUUUUCU | AA | AUACCCCCCGUAU | A | UUCAAUU**UCC**AAUUGAA | AAGAAAA |  | AGAAAAUA |
|  | *Tetranychus phaselus* | AUUUUCU | AA | AUACCCCCCGUAU | A | UUCAAUU**UCC**AAUUGAA | AAGAUUUA |  | AGAAAAUA |
|  | *Tetranychus pueraricola* | AUUUUCU | AA | AUACCCCCCGUAU | A | UUCAAUU**UCC**AAUUGAA | AAGAAUA |  | AGAAAAUA |
|  | *Tetranychus urticae* Green | AUUUUCU | AA | AUACCCCCCGUAU | A | UUCAAUU**UCC**AAUUGAA | AAGAAUA |  | AGAAAAUA |
|  | *Tetranychus urticae* Red | AUUUUCU | AA | AUACCCCCCGUAU | A | UUCAAUU**UCC**AAUUGAA | AAGAAUA |  | AGAAAAUA |
| *trnT* | *Tetranychus kanzawai* | AUUUUA | UUAUU | UUAAGUUUGUAA | A | UAAAUUU**UGU**AAAUUUA | UUUUUU |  | UAAAACU |
|  | *Tetranychus ludeni* | AUUUUA | UUAAU | UUAAGUUAAAUAA | A | UAAAUUU**UGU**AAAUUUA | UUUUUUU |  | UAAAAUA |
|  | *Tetranychus malaysiensis* | AUUUUA | UUA | UUAUAAGUUUAUAA | AA | UAAAUUU**UGU**AAAUUUA | UUUUUUU |  | UAAAACU |
|  | *Tetranychus phaselus* | AUUUUA | UUAUU | UUAAGUUAAGUAA | A | UAAAUUU**UGU**AAAUUUA | UUUUU |  | UAAAAAU |
|  | *Tetranychus pueraricola* | AUCUUA | UUACU | UUAAGUUUAUAA | A | UAAAUUU**UGU**AAAUUUA | UUUUUU |  | UAAAACU |
|  | *Tetranychus urticae* Green | AUUUUA | UUAUU | UUAAGUUAAUAA | A | UAAAUUU**UGU**AAAUUUA | UUUUUU |  | UAAAACU |
|  | *Tetranychus urticae* Red | AUUUUA | UUAUU | UUAAGUUAAUAA | A | UAAAUUU**UGU**AAAUUUA | UUUUUU |  | UAAAGCU |
| *trnL2* | *Tetranychus kanzawai* | UUCAAAA | AA | GCAAAAUGC | A | UAAAAUU**UAA**GAUUUUA | UAUU | GUGGAUAUAUUCAC | UUUUGAAA |
|  | *Tetranychus ludeni* | UUCAAAA | AA | GCAAAUUGC | A | UAAAAUU**UAA**GAUUUUA | UUUU | GUGAAUUACUUCAC | UUUUGAAA |
|  | *Tetranychus malaysiensis* | UUCAAAA | AA | GCAAAUUGC | G | UAAAAUU**UAA**AAUUUUA | UAUU | AUGAAUAAACUCAU | UUUUGAAA |
|  | *Tetranychus phaselus* | UUCAAAA | AA | GCAAAAUGC | A | UAAAAUU**UAA**GAUUUUA | UUUA | GUGUAAAACAC | UUUUGAAA |
|  | *Tetranychus pueraricola* | UUCAAAA | AA | GCAAAUUGC | A | UAAAAUU**UAA**GAUUUUA | UAUU | GUGAAUAUUUUUUCAC | UUUUGAAA |
|  | *Tetranychus urticae* Green | UUCAAAA | AA | GCAAAAUGC | A | UAAAAUU**UAA**GAUUUUA | UAUU | GUGAAUAUUUUCAC | UUUUGAAA |
|  | *Tetranychus urticae* Red | UUCAAAA | AA | GCAAAAUGC | A | UAAAAUU**UAA**GAUUUUA | UAUU | GUGAAUAUUUUCAC | UUUUGAAA |
| *trnQ* | *Tetranychus kanzawai* | GUUUAAA | UUUUUUU |  |  | AAGUUU**UUG**AAACUU | UUU | AAUUUUUU | UUCAAAAA |
|  | *Tetranychus ludeni* | GUUUAAA | UUUUU |  |  | AAAGUUU**UUG**AAACUUU | UUUUUGUUU |  | UUCAAAAA |
|  | *Tetranychus malaysiensis* | GUUUAAA | UAUAA |  |  | AAAGUUU**UUG**AAACUUU | AAGUUUAUUU |  | UUCAAAAA |
|  | *Tetranychus phaselus* | GUUUAAA | CUUUUU |  |  | AAGUUU**UUG**AAACUU | UAUUUUUUUU |  | UUCAAAAA |
|  | *Tetranychus pueraricola* | GUUUAAA | UUUUUA |  |  | AAAGUUU**UUG**AAACUUU | UUUUUAUUU |  | UUCAAAAA |
|  | *Tetranychus urticae* Green | GUUUAAA | AUUAUU |  |  | UAAGUUU**UUG**AAACUUA | UUUUUAUUU |  | UUCAAAAA |
|  | *Tetranychus urticae* Red | GUUUAAA | UUUCUU |  |  | UAAGUUU**UUG**AAACUUA | UUCUUAUUU |  | UUCAAAAA |
| *trnC* | *Tetranychus kanzawai* | UGAUAGA | GGA | AACCCUU | U | UUUGUUU**GCA**ACACAAA | AAAUAU |  | UCUAUCAA |
|  | *Tetranychus ludeni* | UGAUAG | AGGAA | AACACCCUU | U | UUUGUUU**GCA**AUACAAA | AAUAUUA |  | UUUUCUA |
|  | *Tetranychus malaysiensis* | UGAUAG | AGGAAAA | UACCAACA | U | UUUGUUU**GCA**ACACAAA | AAUAC |  | CUAUCAA |
|  | *Tetranychus phaselus* | UGAUAGA | GGUAA | AAUACCAUU | A | UUUGUUU**GCA**ACACAAA | AAUUU |  | UCUAUCAA |
|  | *Tetranychus pueraricola* | UGAUAGA | GGAAA | AACCUU | U | UUUGUUU**GCA**ACACAAA | AAACUU |  | UCUAUCAA |
|  | *Tetranychus urticae* Green | UGAUAGA | GGAAA | AACCUU | U | UUUGUUU**GCA**ACACAAA | AAUAAU |  | UCUAUCAA |
|  | *Tetranychus urticae* Red | UGAUAGA | GGAA | AACCUU | U | UUUGUUU**GCA**ACACAAA | AAUAAU |  | UCUAUCAA |
| *trnS2* | *Tetranychus kanzawai* | AAAGU | UAAAAA |  |  | UUUAUUU**UGA**AAAUAAA | U | UUCAUUUAA | ACUUUU |
|  | *Tetranychus ludeni* | AAAGU | UAAAAA |  |  | UUUAUUU**UGA**AAAUAAA | UUU | UAUUAUA | ACUUUU |
|  | *Tetranychus malaysiensis* | AAAGU | UAAAAA |  |  | UUUAUUU**UGA**AAAUAAA | UUUA | UUUAAAA | ACUUUU |
|  | *Tetranychus phaselus* | AAAGU | UAAAAAA |  |  | UUUAUUU**UGA**AAAUAAA | UU | UUAUAAA | ACUUUU |
|  | *Tetranychus pueraricola* | AAAGU | UAAAAA |  |  | UUUAUUU**UGA**AAAUAAA | UU | UUAUUCAAA | ACUUUU |
|  | *Tetranychus urticae* Green | AAAGU | UAAAAA |  |  | UUUAUUU**UGA**AAAUAAA | UU | UUAUUCAA | ACUUUU |
|  | *Tetranychus urticae* Red | AAAGU | UAAAAA |  |  | UUUAUUU**UGA**AAAUAAA | UU | UUAUUCAA | ACUUUU |
| *trnA* | *Tetranychus kanzawai* | UUUUAAG | UUAAA | UUUUAA | A | AUAAUUU**UGC**AAAUUAU | UUUUUUU |  | CUUAAAGU |
|  | *Tetranychus ludeni* | UUUUAAG | UACAAAAA |  |  | AUAAUUU**UGC**AAAUUAU | UUUUU |  | CUUAAAGU |
|  | *Tetranychus malaysiensis* | UUUUAAG | UUUA | UUUAAAA | A | AUAAUUU**UGC**AAAUUAU | UAUGUUU |  | CUUAAAGU |
|  | *Tetranychus phaselus* | UUUUAAG | UUAA | UUAAA | A | ACAAUUU**UGC**AAAUUGU | UUUUUU |  | CUUAAAGU |
|  | *Tetranychus pueraricola* | UUUUAAG | UCUAG | UUUAA | A | AUAAUUU**UGC**AAAUUAU | UUUUUUU |  | CUUAAAGU |
|  | *Tetranychus urticae* Green | UUUUAAG | UUGAA | UUUAA | A | AUAAUUU**UGC**AAAUUAU | UUUUU |  | CUUAAAGU |
|  | *Tetranychus urticae* Red | UUUUAAG | UUGAA | UUUAA | A | AUAAUUU**UGC**AAAUUAU | UUUUU |  | CUUAAAGU |
| *trnH* | *Tetranychus kanzawai* | AUUGAA | AUA | GUUUAUAAAAAU | A | UUAAAUU**GUG**AAUUUAA | AG | AAAUAUU | UUCAAUA |
|  | *Tetranychus ludeni* | AUUGGA | AUA | GUUUAUUAAAAAU | A | UUAAAUU**GUG**AAUUUAA | AG | AAAAAU | UUCAAUA |
|  | *Tetranychus malaysiensis* | AUUGAA | AUA | GUUUAAAAAAAAU | A | UUAAAUU**GUG**AAUUUAA | AG | AAAUUU | UUCAAUA |
|  | *Tetranychus phaselus* | AUUGGA | AUA | GUUUAUCUUAAAAU | A | UUAAAUU**GUG**AAUUUAA | AG | AAAAUU | UUCAAUA |
|  | *Tetranychus pueraricola* | AUUGAA | AUA | GUUUAUACAAAAU | A | UUAAAUU**GUG**AAUUUAA | AG | AAAUUU | UUCAAUA |
|  | *Tetranychus urticae* Green | AUUGGA | AUA | GUUUAUACAAAAAU | A | UUAAAUU**GUG**AAUUUAA | AG | AAGUUU | UUCAAUA |
|  | *Tetranychus urticae* Red | AUUGGA | AUA | GUUUAUACAAAAAU | A | UUAAAUU**GUG**AAUUUAA | AG | AAGUUU | UUCAAUA |
| *trnW* | *Tetranychus kanzawai* | ACUAAAU | UUA | AGUUAUAUUAAACU | A | UUUACUU**UCA**AAGUAAA | AU | AAUUU | AUUUAGAA |
|  | *Tetranychus ludeni* | TCUAAAU | UUA | AGUUAUAUUAAACU | A | UUUACUU**UCA**AAGUAAA | AU | AAUUU | AUUUAGAA |
|  | *Tetranychus malaysiensis* | TCUAAAU | UUA | AGUUAUACUAAACU | A | UUUACUU**UCA**AAGUAAA | AU | AAUUU | AUUUAGAA |
|  | *Tetranychus phaselus* | ACUAAAU | UUA | AGUUAUUUAAACU | A | UUUACUU**UCA**AAGUAAA | AU | AAUUU | AUUUAGAA |
|  | *Tetranychus pueraricola* | ACUAAAU | UUA | AGUUAUAUUAAACU | A | UUUACUU**UCA**AAGUAAA | AU | AAUUU | AUUUAGAA |
|  | *Tetranychus urticae* Green | ACUAAAU | UUA | AGUUAUAUAAAACU | A | UUUACUU**UCA**AAGUAAA | AU | AAUUU | AUUUAGAA |
|  | *Tetranychus urticae* Red | ACUAAAU | UUA | AGUUAUAUUAAACU | A | UUUACUU**UCA**AAGUAAA | AU | AAUUU | AUUUAGAA |
| *trnM* | *Tetranychus kanzawai* | UUAGG | AUUA | AGCUAUAAAAGCU | A | AAGGAUU**CAU**AUUCCUU | UG | AAUUUUU | CCUAAA |
|  | *Tetranychus ludeni* | UUAGG | AUUA | AGCUAUAUAAAGCU | A | AAGGAUU**CAU**AUUCCUU | UGA | AUUUAU | CCUAAA |
|  | *Tetranychus malaysiensis* | UUAGGG | AUA | AGCUAUUAUUUAAGCU | A | AAGGACU**CAU**AAUCCUU | UGAAUUUA |  | CCCUAAA |
|  | *Tetranychus phaselus* | UUAGG | AGUA | AGCUAUAUAAAGCU | A | AAGGAUU**CAU**AUUCCUU | UG | AAUUUUU | CCUAAA |
|  | *Tetranychus pueraricola* | UUAGG | AGUA | AGCUAUAAAAGCU | A | AAGGAUU**CAU**AUUCCUU | UG | AAUUUUU | CCUAAA |
|  | *Tetranychus urticae* Green | UUAGG | AGUA | AGCUAUAUAAGCU | A | AAGGAUU**CAU**AUUCCUU | UG | AAUUUUU | CCUAAA |
|  | *Tetranychus urticae* Red | UUAGG | AGUA | AGCUAUAUAAGCU | A | AAGGAUU**CAU**AUUCCUU | UG | AAUUUUU | CCUAAA |
| *trnS1* | *Tetranychus kanzawai* | AAGAAA | AUAUUU |  |  | UAAAGCU**GCU**AACUUUA | AAU | UAAAUUA | UUUCUUU |
|  | *Tetranychus ludeni* | AAGAAA | AUAUCU |  |  | UAAAGCU**GCU**AACUUUA | AAU | UAAAUUA | UUUCUUU |
|  | *Tetranychus malaysiensis* | AAGAAA | AAUUU |  |  | UAAAGCU**GCU**AACUUUA | AACAAAAUAA |  | UUUCUUU |
|  | *Tetranychus phaselus* | AAGAAA | ACAUUU |  |  | UAAAGCU**GCU**AACUUUA | AAUU | UAUUUA | UUUCUUU |
|  | *Tetranychus pueraricola* | AAGAAA | AAACUU |  |  | UAAAGCU**GCU**AACUUUA | AA | UUAAAUAA | UUUCUUU |
|  | *Tetranychus urticae* Green | AAGAAA | AUAUUU |  |  | UAAAGCU**GCU**AACUUUA | AAU | UUAAUAA | UUUCUUU |
|  | *Tetranychus urticae* Red | AAGAAA | AUAUUU |  |  | UAAAGCU**GCU**AACUUUA | AA | UUAAAUAA | UUUCUUU |
| *trnV* | *Tetranychus kanzawai* | CUCUAA | UUUAA | UUAAA | A | UAAAAUU**UAC**AAUUUUA | AUUUUUU |  | UUAGAAA |
|  | *Tetranychus ludeni* | CUCUAA | UUUU | AUAAAU | A | UAAAAUU**UAC**AGUUUUA | UCUUUA |  | UUAGAAA |
|  | *Tetranychus malaysiensis* | CUCUAA | AUUU | AAUAUU | A | AAAAUU**UAC**AAUUUU | AUAUAGU |  | UUAGAAA |
|  | *Tetranychus phaselus* | CUCUAA | UGAAA | UUAAA | A | UAAAAUU**UAC**AAUUUUA | AUUAUA |  | UUAGAAA |
|  | *Tetranychus pueraricola* | CUCUAA | UUUUA | UUAAA | A | UAAAAUU**UAC**AAUUUUA | AUUUUA |  | UUAGAAA |
|  | *Tetranychus urticae* Green | CUCUAA | UUUUA | UUAAAA | A | UAAAAUU**UAC**AAUUUUA | AUUUUA |  | UUAGAAA |
|  | *Tetranychus urticae* Red | CUCUAA | UUUUA | UUAAAA | A | UAAAAUU**UAC**AAUUUUA | AUUUUA |  | UUAGAAA |
| *trnI* | *Tetranychus kanzawai* | AGUAA | UAUUU |  |  | AGUUAUU**GAU**AAUUAACU | UUAAAAAAAU |  | UUACUA |
|  | *Tetranychus ludeni* | AGUAAU | GUUU |  |  | AGUUAUU**GAU**AAUUAACU | UAAAUAAA |  | AUUACUA |
|  | *Tetranychus malaysiensis* | AGUAAA | AUUU |  |  | AGUUAUU**GAU**AAUUAACU | UUUUUCAGA |  | UUUACUA |
|  | *Tetranychus phaselus* | AGUAAU | AUUUU |  |  | AGUUAUU**GAU**AAUUAACU | UACCAAAA |  | AUUACUA |
|  | *Tetranychus pueraricola* | AGUAA | AAUUU |  |  | AGUUAUU**GAU**AAUUAACU | UUUAAAAAC |  | UUACUA |
|  | *Tetranychus urticae* Green | AGUAA | UAUUU |  |  | AGUUAUU**GAU**AAUUAACU | UUCAGAAAU |  | UUACUA |
|  | *Tetranychus urticae* Red | AGUAAA | AUUU |  |  | AGUUAUU**GAU**AAUUAACU | UUUAGAAA |  | UUUACUA |

Putatively-paired nucleotides are underlined. Anticodon bases are bold text.
