# Supplementary material for: The Complete Mitochondrial Genomes of Six Species of Tetranychus Provide Insights into the Phylogeny and Evolution of Spider Mites
Source: PLoS One. 2014 Oct 16;9(10):e110625. doi: 10.1371/journal.pone.0110625 (PMC4199730; doi:10.1371/journal.pone.0110625)
Supplement: Table S6 — Pairwise distance between mitochondrial genomes. (DOC) [file pone.0110625.s014.doc]

**Table S6. Pairwise distance between mitochondrial genomes.**

|  | *Tu* Green | | *Tu* Red | | *Tk* | | *Tl* | | *Tm* | | *Tpa* | |
| --- | --- | --- | --- | --- | --- | --- | --- | --- | --- | --- | --- | --- |
| *Tetranychus urticae* Green | Dist^a^ | Std. Err^b^ | Dist | Std. Err | Dist | Std. Err | Dist | Std. Err | Dist | Std. Err | Dist | Std. Err |
| *Tetranychus urticae* Red | 0.027 | 0.002 |  |  |  |  |  |  |  |  |  |  |
| *Tetranychus kanzawai* | 0.089 | 0.002 | 0.089 | 0.002 |  |  |  |  |  |  |  |  |
| *Tetranychus ludeni* | 0.172 | 0.004 | 0.171 | 0.004 | 0.175 | 0.004 |  |  |  |  |  |  |
| *Tetranychus malaysiensis* | 0.209 | 0.003 | 0.209 | 0.003 | 0.212 | 0.004 | 0.211 | 0.004 |  |  |  |  |
| *Tetranychus phaselus* | 0.166 | 0.003 | 0.168 | 0.003 | 0.168 | 0.004 | 0.169 | 0.003 | 0.202 | 0.004 |  |  |
| *Tetranychus pueraricola* | 0.081 | 0.003 | 0.082 | 0.003 | 0.092 | 0.003 | 0.175 | 0.004 | 0.205 | 0.004 | 0.167 | 0.004 |

^a^ Dist = distance, analyses were calculated using Kimura 2-parameter model. ^b^ Std. Err = Standard error, estimations were obtained by bootstrapping 1000 replicates.
